# The transcriptional landscape of atrial fibrillation: A systematic review and meta-analysis

**DOI:** 10.1371/journal.pone.0323534

**Published:** 2025-05-30

**Authors:** Sergio Alejandro Gómez-Ochoa, Malte Möhn, Michelle Victoria Malz, Roger Ottenheijm, Jan D. Lanzer, Felix Wiedmann, Manuel Kraft, Taulant Muka, Constanze Schmidt, Marc Freichel, Rebecca T. Levinson

**Affiliations:** 1 Department of General Internal Medicine and Psychosomatics, Heidelberg University Hospital, University of Heidelberg, Heidelberg, Germany; 2 Institute for Computational Biomedicine, Heidelberg University Faculty of Medicine, Heidelberg University Hospital, University of Heidelberg, Heidelberg, Germany; 3 Institute of Pharmacology, Heidelberg University, Heidelberg, Germany; 4 Department of Cardiology, Heidelberg University Hospital, University of Heidelberg, Heidelberg, Germany; 5 DZHK (German Center for Cardiovascular Research), Partner Site Heidelberg/Mannheim, University of Heidelberg, Heidelberg, Germany; 6 HCR, Heidelberg Center for Heart Rhythm Disorders, University of Heidelberg, Heidelberg, Germany,; 7 Epistudia, Bern, Switzerland.; University of Ilorin, NIGERIA

## Abstract

**Background:**

Despite advances in understanding atrial fibrillation (AF) pathophysiology, there is limited agreement on the key genes driving its pathophysiology. To understand the genome-wide transcriptomic landscape, we performed a meta-analysis from studies reporting gene expression patterns in atrial heart tissue from patients with AF and controls in sinus rhythm (SR).

**Methods:**

Bibliographic databases and data repositories were systematically searched for studies reporting gene expression patterns in atrial heart auricle tissue from patients with AF and controls in sinus rhythm. We calculated the pooled differences in individual gene expression from fourteen studies comprising 534 samples (353 AF and 181 SR) to create a consensus signature (CS), from which we identified differentially regulated pathways, estimated transcription factor activity, and evaluated its performance in classifying validation samples as AF or SR.

**Results:**

Despite heterogeneity in the top differentially expressed genes across studies, the AF-CS in both chambers were robust, showing a better performance in classifying AF status than individual study signatures. Functional analysis revealed commonality in the dysregulated cellular processes between chambers, including extracellular matrix remodeling (highlighting epithelial mesenchymal transition, actin filament organization, and actin binding hallmark pathways), cardiac conduction (including cardiac muscle cell action potential, gated channel activity, and cation channel activity pathways), metabolic derangements (highlighting oxidative phosphorylation and asparagine n linked glycosylation), and innate immune system activity (mainly neutrophil degranulation, and TNFα signaling pathways). Finally, the AF-CS showed a good performance differentiating AF from controls in three validation datasets (two from peripheral blood and one from left ventricle samples).

**Conclusions:**

Despite variability in individual studies, this meta-analysis elucidated conserved molecular pathways involved in AF pathophysiology across its phenotypes and the potential of a transcriptomic signature in identifying AF from peripheral blood samples. Our work highlights the value of integrating published transcriptomics data in AF and the need for better data deposition practices.

## Introduction

Atrial fibrillation (AF) is the most prevalent sustained arrhythmia worldwide [1]. It is associated with substantial quality of life deterioration and a high risk of adverse clinical outcomes, including thromboembolic events and mortality [2]. AF is a heterogeneous disease that arises in combination with other cardiovascular diseases, such as hypertension and heart failure, and significantly contributes to chronic disease burden on the individual and the healthcare system [3,4]. Despite the broad array of available diagnostic tools and therapeutic interventions, recurrent AF, treatment resistance, and adverse side effects of current pharmacotherapies remain pressing challenges [5]. Moreover, current therapies do not explicitly target AF’s molecular and genetic contributors, potentially limiting the possibility of optimally managing this condition [6]. Therefore, a more complete understanding of AFs underlying molecular and genetic contributors has become essential [7].

Transcriptomic analyses have emerged as a valuable tool for probing AF’s genetic and molecular foundations [8]. However, a significant gap in the existing research landscape is the lack of comprehensive studies considering the entire transcriptome [9–13]. When studies analyze the complete transcriptome, they often still focus on identifying specific genes, which may vary widely between studies, resulting in a high degree of heterogeneity in the key AF genes identified [14]. Technical differences between experiments may partially explain these differences; however, contrasts in sociodemographic and clinical features, such as age, gender, ethnicity, comorbidities, and AF type, between cohorts also represent a relevant source of variation in the observed trends [15]. Consequently, these factors may inadvertently influence the gene expression profiles associated with disease, result in patient- or population-specific genomic responses, and affect the expression levels of genes involved in the etiology of AF [16]. A consensus transcriptional signature, which could provide a more unified perspective on the molecular processes that instigate and perpetuate this disorder, could offer relevant insights into the disease and lay the groundwork for more personalized and effective treatment strategies [17].

This consensus signature meta-analytical approach using transcriptomic data has been previously applied in studies evaluating cardiac tissue from patients with heart failure (HF) and renal tissue from patients with chronic kidney disease, validating the feasibility of combining RNA-sequencing and microarray data from different sources, platforms, and conditions, as well as providing a statistical framework applicable in other contexts [18,19]. In light of this, the primary aim of our study was to develop a consensus transcriptional signature for the left- and right atrial appendages in AF. To accomplish this, we undertook a systematic review and meta-analysis of existing studies comparing myocardial gene expression patterns in patients diagnosed with AF and control subjects, presumably in sinus rhythm (SR). Moreover, to understand the translational relevance of this signature, we aimed to assess whether this consensus signature can be leveraged in other scenarios, such as peripheral blood transcriptomics, to accurately differentiate patients with AF from controls. This consensus signature represents an open tool easily accessible by the research community with the potential of usage without significant previous data analysis experience.

## Methods

### Search strategy and selection criteria.

We followed two recent guidelines for the conduct of the systematic review and meta-analysis and Preferred Reporting Items for Systematic Reviews and Meta-Analyses (PRISMA) guidelines for reporting (S1 Table) [20–22]. The protocol of this study is registered in the PROSPERO database with the record code CRD42023399021.

MEDLINE/Ovid, EMBASE/Ovid, CINAHL/EBSCOhost, and Google Scholar were searched to identify relevant studies from January 1st, 2000, until March 18th, 2024, without language restrictions. Search terms related to AF transcriptomics were used, including atrial fibrillation, atrial cardiomyopathy, transcriptome, microarrays, and RNA-Seq, among many others. The complete search strategy is described in S1 Text. All observational studies comparing the transcriptomic profile (by microarrays or RNA-Seq) of patients with a diagnosis of AF with individuals suspectedly in SR were included. Only studies that assessed differential gene expression between AF and SR groups by analysis of myocardial tissue samples and provided any type of dataset suitable for reanalysis were included.

In parallel to the bibliometric database search, we queried four additional repositories that store biomedical studies datasets: the Gene Expression Omnibus database, the European Nucleotide Archive, ArrayExpress, and the European Genome Phenome Archive. The following search terms were used: “atrial fibrillation,” “atrial cardiomyopathy,” “atrial arrhythmia,” and “afib.” We included a dataset if it assessed myocardium samples, if data from at least four total patients were available, and if there was a protocol describing sample gathering and processing methodology available. S1 Fig summarizes the main steps in the analysis process of this study [18,19].

### Database screening and data extraction

Four reviewers screened titles and abstracts in duplicate to systematically identify potentially includable studies according to the selection criteria. Subsequently, four reviewers independently extracted the following data from each included study: name of the first author, the country in which the study was performed, year of publication, study design, the total number of patients, number per group (AF vs. SR), demographic characteristics of the population evaluated, type of AF evaluated, left ventricular ejection fraction, sample sources, sequencing and analysis methods, and differentially expressed genes between groups.

### Quality assessment

Four authors independently assessed each study in duplicate using an adapted version of the Checklist for Analytical Cross-Sectional Studies from the Joanna Briggs Institute (JBI). This adapted checklist was designed due to the lack of specific quality assessment tools for studies performing unbiased transcriptomic analysis in tissue samples and allowed an objective assessment of relevant methodological and reporting aspects of unique importance in this type of studies. It comprises ten items covering study design and participant characterization, sample processing, statistical analysis, and data reporting. Conflicts were resolved by consensus or the intervention of an additional reviewer.

### Data processing

The raw data from studies found through the systematic search strategy and from queried repositories were downloaded and processed uniformly. Non-normalized count matrices were downloaded directly, whereas, for studies with only sequencing files (.fastq files), sequence alignment was performed using the HISAT2 alignment algorithm to acquire count matrices for data derived from RNA-Seq experiments. Standard processing and normalization were performed using the limma package for datasets derived from RNA-seq, while the oligo package was also used for those derived from microarrays.

### Sample classification and contrasts

We classified the collected samples according to AF rhythm (AF vs. SR) and according to sample origin (left atrial appendage and right atrial appendage [LAA and RAA, respectively]). From this, the differential gene expression between AF and SR in each chamber was analyzed.

### Statistical analysis

Overall, a consensus transcriptional signature meta-analysis can be defined as an approach to identify consistent gene expression patterns (transcriptional signatures) across multiple independent studies focusing on a particular condition [18,19]. This approach allows for the identification of more robust and reliable molecular signatures, potentially elucidating pivotal transcriptional programs consistently correlated with specific states or responses, such as disease onset, progression, or reaction to specific interventions. For this purpose, a Fisher’s combined probability test was used to pool the differential expression analysis results per individual genes if they were evaluated in at least 50% of the studies. For each contrast, the degrees of freedom for the statistical tests were derived from the number of studies included. Since none of the included studies mentioned having performed any probabilistic sampling procedure, additional study weighting was not performed. After the Benjamini-Hochberg (BH) correction of the pooled P values, a gene ranking representing the AF transcriptomic signature was generated [23]. Using an inferential approach, we estimated the accuracy of using individual studies to classify samples of other studies into either “AF” or “SR” categories. For this purpose, a disease score was calculated by linearly combining the t-values of the differentially expressed genes of the reference studies (disease pattern) with their expression values in the individual test study.

The number of disease classifiers built was the same as the total study number for each comparison based on the t-values of the differentially expressed genes in each contrast. Once each sample-level disease score was calculated, the area under the receiver operating characteristic curve (AUROC) was used to test the score’s accuracy to discriminate samples from AF patients, reflecting the conservation of gene regulation patterns across studies. Finally, the consistency of the direction of transcriptional regulation in the disease score results was also assessed by separating the top differentially expressed genes based on their regulation direction and performing gene set enrichment analysis (GSEA).

In addition, we assessed the concordance of the top 500 genes of each chamber’s consensus signature using the Jaccard index. Complementarily, we compared the consensus rankings of LAA and RAA using the Rank-Rank Hypergeometric Overlap (RRHO) test. This threshold-free algorithm detects common trends between two ordered lists of differentially expressed genes. In this method, the genes are ordered based on the product of the negative logarithm (base 10) of the differential expression p-value and the effect size direction [24]. According to this ranking, genes with unchanged, upstream, and downstream effects are positioned in the middle, at the top, and at the bottom of the list, respectively. The degree of similarity between the gene lists from two different datasets is then quantified through a one-sided p-value, which is determined using the principles of the hypergeometric distribution. Missing data were not imputed.

### Sensitivity analysis

Using a leave-one-out procedure, we evaluated how much each study impacted the overall findings. Therefore, the meta-analysis for each contrast was repeated the same number of times of the amount of studies present, each time leaving out the data from one study. After each round of analysis, we performed a Spearman rank correlation test comparing the leave-one-out result with the full meta-analysis to test the influence of the left-out study. A correlation value close to 1 would indicate that the results were almost identical, and therefore, the excluded study did not significantly impact the overall findings. Conversely, a low correlation value would highlight the studies that strongly influenced the meta-analysis. This approach allowed us to assess the robustness of the meta-analysis ranking rigorously.

### Added value of the meta-analysis

To analyze the benefit of combining the available transcriptomic data related to AF in our meta-analysis, we examined whether the top 500 genes identified from the consensus signature provided a more representative transcriptional ‘fingerprint’ of AF than the top 500 genes picked from each study. In particular, we checked whether the AUROCs based on the consensus genes were significantly better than those derived from individual studies using the Wilcoxon paired test. At first, we divided the top 500 genes into two groups: those up- and down-regulated in AF. We then checked for their presence in each individual study’s gene activity ranking using GSEA. Then, we compared these enrichment scores to the ones obtained using the top 500 genes from each separate study with the Wilcoxon paired test.

### Functional analysis

After the meta-analysis, each gene’s resulting -log10 p-value was weighted based on the mean direction of change across the studies to develop the directed consensus signature. To comprehensively characterize the biological processes associated with AF, we performed multiple complementary functional analyses. First, we conducted gene set enrichment analysis using the Molecular Signatures Database (MSigDB) to identify enriched gene ontology terms, pathways, and biological processes. This was implemented using the *msigdbr* package with a ranked list of genes ordered by their weighted -log10 p-values. We tested for enrichment against multiple MSigDB collections, including GO biological processes, molecular functions, cellular components, Hallmark gene sets, KEGG, and Reactome pathways. In parallel, we estimated transcription factor (TF) activity using DoRothEA, which infers TF activity based on the expression changes of their known target genes [25–27]. Furthermore, we analyzed signaling pathway activity using PROGENy, which estimates pathway activities based on pathway-responsive genes [28]. For all analyses, we applied the Benjamini-Hochberg (BH) procedure to adjust p-values for multiple testing, considering results significant at an adjusted p-value < 0.05.

### Tissue-cell-type enrichment of the AF-CS

Using the differentially expressed genes (BH p-value < 0.05) in each CS, we aimed to explore the potential cellular context in each chamber using WebCSEA. This tool provides a specialized query interface for analyzing gene sets against an extensive repository of cell expression profiles specific to various human tissue types. For each query run, the system produces a series of analytical metrics, including the raw statistical significance value (p-value) specific to cell type enrichment analysis (CSEA), a consolidated p-value derived using a permutation-based statistical approach, and a list of shared genes between the query set and the cell type-tissue-specific expression profiles.

### Performance of the AF-CS as predictors of AF status

Finally, we aimed to explore how the two consensus transcriptional signatures derived from atrial myocardium analyses would perform in classifying other types of samples (peripheral blood, left ventricle and epicardial adipose tissue) derived from AF and SR patients in publicly available datasets.

### Study registration

The study was registered in the International Prospective Register of Systematic Reviews (PROSPERO) under the record code CRD42023399021. Available from: https://www.crd.york.ac.uk/prospero/display_record.php?ID=CRD42023399021.

## Results

### Descriptive analysis

**Fig 1:**
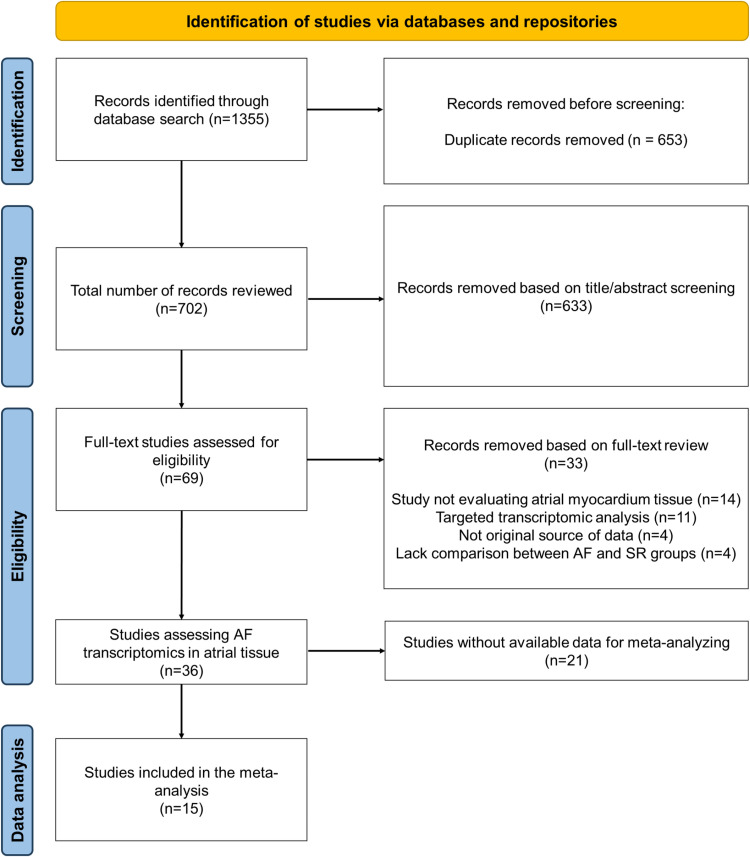
Flowchart of study selection.

This systematic review and meta-analysis was performed in compliance with Preferred Reporting Items for Systematic Reviews and Meta-Analyses (PRISMA) guidelines for reporting (S1 Table). Of the 1243 references screened, we identified 36 studies that applied transcriptomic approaches in atrial tissue for the study of AF (S2 Table) [13,29–61]. Of these, only 15 studies met the complete selection criteria by providing complete gene counts or raw sequences for each of the assessed samples, therefore being included in the meta-analysis (Fig 1) [29,31,34,35,37–39,48–52,56,61,62].

**Fig 2:**
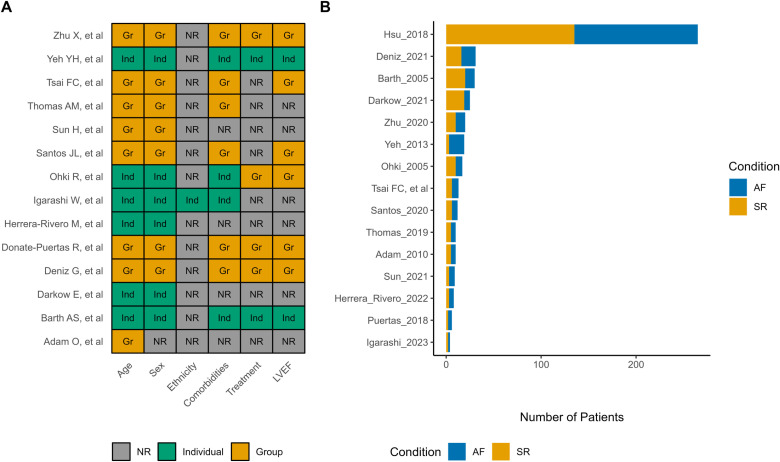
Summary of overall study characteristics. A, Available metadata on relevant characteristics in the included datasets. B, Sample size per group categories. *Abbreviations.* Ind: Individual-patient data; Gr: Group data (measures of central tendency and dispersion); NR: Not reported.

The selected studies were published between 2005 and 2023, with the majority (9 [60%] of 15) published within the past five years. The data sets derived from these studies consisted of a total of 534 samples (353 AF and 181 SR) from 470 patients (mean age 58.5 years; 58% male) (S2 Fig and S3 Table) [48,63–75]. Most samples were collected in the context of valve surgery (14/15; 93%), followed by coronary artery bypass grafting (CABG) surgery (7/15; 47%) and cardiac transplantation (2/15; 13%). The majority of studies included patients with permanent or persistent AF, with only three including patients with paroxysmal AF. Nevertheless, the lack of individual-patient information limited the assessment of different AF types independently (Table 1). Fig 2 summarizes the general characteristics of the studies included in the meta-analysis, explicitly highlighting the reporting of relevant information (Fig 2A) and the sample sizes evaluated (Fig 2B). Unfortunately, only a small minority of studies reported individual information on the included patients, which limited the performance of analyses by relevant covariates. From the total studies, 11 analyzed samples from the left atrial appendage (LAA), while eight analyzed samples from the right atrial appendage (RAA) (Table 1). Finally, both microarray (n = 7; 47%) and RNA-Seq (n = 8; 53%) technologies were utilized (Table 1).

**Table 1:** General characteristics of the included studies.

First Author	Year	Country	Total Sample	AF Patients	SR Patients	AF Types Evaluated	Sample Collection Context	Sample Sources	Technology	Platform
Adam O, et al [29]	2010	Germany	10	5	5	Permanent	Valve replacement surgery	Left atrial appendage	Microarrays	Affymetrix HGU133-Plus 2.0
Barth AS, et al [31]	2005	Germany	30	10	20	Permanent	Valve replacement surgery; Coronary Artery Bypass Graft surgery	Right atrial appendage	Microarrays	Affymetrix Human Genome U133A Array
Darkow E, et al [34]	2021	Germany	25	6	19	Permanent	Valve replacement surgery; Coronary Artery Bypass Graft surgery	Right atrial appendage	RNA-Seq	Illumina HiSeq4000
Deniz G, et al [35]	2021	Turkey	31	15	16	Persistent	Valve replacement surgery	Left atrial appendage; Right atrial appendage	Microarrays	Affymetrix HGU133-Plus 2.0
Doñate-Puertas R, et al [37]	2017	France	6	4	2	Paroxysmal; Persistent; Permanent	Valve replacement surgery	Left atrial appendage	Microarrays	Human Genome U133A 2.0 Array
Herrera-Rivero M, et al [38]	2022	Netherlands	8	5	3	Paroxysmal; Persistent	Valve replacement surgery; Coronary Artery Bypass Graft surgery; Heart transplantation	Left atrial appendage; Right atrial appendage; Ventricles	RNA-Seq	Illumina NextSeq500
Hsu J, et al. [39]	2018	USA	265	130	135	Not reported	Valve replacement surgery; Coronary Artery Bypass Graft surgery	Left atrial appendage	RNA-Seq	Illumina HiSeq 2000
Igarashi W, et al. [62]	2023	Japan	4	2	2	Permanent	Valve replacement surgery	Right atrial appendage	RNA-Seq	Illumina NovaSeq 6000
Ohki R, et al [48]	2005	Japan	17	7	10	Not reported	Valve replacement surgery; Coronary Artery Bypass Graft surgery	Right atrial appendage	Microarrays	Human Genome U95A Set
Santos JL, et al [49]	2020	Denmark	12	6	6	Persistent; Permanent	Valve replacement surgery	Right atrial appendage	RNA-Seq	Illumina HiSeq 2500
Sun H, et al [50]	2021	China	9	6	3	Paroxysmal; Persistent	Heart transplantation	Left atrial appendage	RNA-Seq	Illumina HiSeq X ten
Thomas A, et al [51]	2019	England	10	5	5	Permanent	Valve replacement surgery; Coronary Artery Bypass Graft surgery	Left atrial appendage; Right atrial appendage	RNA-Seq	Illumina NextSeq500
Tsai F, et al [52]	2016	Taiwan	13	7	6	Persistent	Valve replacement surgery; Coronary Artery Bypass Graft surgery	Left atrial appendage; Right atrial appendage	Microarrays	Affymetrix HGU133-Plus 2.0
Yeh Y, et al [56]	2013	Taiwan	19	16	3	Persistent	Valve replacement surgery	Left atrial appendage; Other	Microarrays	Human Genome U133A 2.0 Array
Zhu X, et al [61]	2020	China	20	10	10	Persistent	Valve replacement surgery	Left atrial appendage	RNA-Seq	Illumina HiSeqTM 2500

Basic characteristics of studies included in the meta-analysis.

To identify bias in the included studies, we used a modified version of the Checklist for Analytical Cross-Sectional Studies from the Joanna Briggs Institute (JBI) which included 10 points (S4 Table). We observed a mean score of 7/10 points for the included studies, with no studies scoring less than four points (S4 Table). The following sections describe the AF-CS results for each evaluated cardiac chamber (L-CS for the LAA and R-CS for the RAA).

### Overall characteristics and consistency measures

At first, we sought to characterize the technical characteristics of the different datasets by atrial appendage. Gene coverage analysis, which assesses the similarities between the assessed genes across studies, highlighted higher comparability of the LAA samples (mean Jaccard index of ≈0.70) than RAA samples (mean Jaccard index of ≈0.51), meaning that overall the LAA sequencing results in the different studies shared more genes between them than the RAA ones (S3 Fig). Moreover, an ANOVA test revealed a significant association between the technology type (“RNA-Seq” vs. “Microarrays”) and the first three PCs of the z-transformed samples (P < 0.05 for the first three PCs in both atria) (S4 Fig). Though, after performing read count standardization, the heterogeneity derived from technical aspects was reduced. Furthermore, we evaluated the proportion of variance explained by age, sex, and left ventricular ejection fraction (LVEF) in standardized PCA to explore the role of clinical and sociodemographic parameters in gene expression patterns. Considering that LVEF represents the most consistently reported echocardiographic parameter and has a direct relationship with atrial function, it was decided to utilize this measure in the absence of more specific parameters for atrial status. We observed that the proportion of men in the different groups was not associated with any principal component, explaining zero percent of the variance. Mean age in each group (AF and SR) explained 3.5% of the variance in LAA and 4.9% in RAA, while mean LVEF explained 4.5% in LAA and 15.3% in RAA. In comparison, the variance explained by AF diagnosis was 7.9% in the LAA and 13% in the RAA (S5 Fig).

We then evaluated the consistency of the results of the studies for genes that ranked highest after differential expression analysis in each individual study (S6 Fig). We compared the top 500 differentially-expressed genes (DEG) in each individual study, observing low concordance in the results of both cardiac chambers (mean Jaccard Index LAA: 0.02 and RAA: 0.03) (S7A and S7E Figs). However, when evaluating the predictive capacity of these top 500 genes of each study to discriminate the diagnosis of AF in other datasets using a disease score, we observed a relatively high performance according to the median AUROC values (LAA: 0.74 and RAA: 0.78) (S7B and S7F Figs). This disease score was calculated by linearly combining the t-values of the differentially expressed genes of the reference studies (disease pattern) with their expression values in the individual test study. This was built assuming that if two or more studies share AF transcriptional signatures, the disease score derived from these could be used to discriminate between sample groups in other independent datasets accurately. Therefore, this disease score would estimate how similar the expression profile observed in each sample with the disease phenotype is, going beyond changes in the mean expression of specific genes and focusing on the joint regulation of multiple genes.

As the molecular responses showed some degree of uniformity across the evaluated studies, we proceeded further and examined whether the direction of differential regulation of the top DEG in each dataset was consistent with their observed trends in the remaining studies. For this purpose, an independent enrichment analysis was performed on the gene-level statistics derived from the top 500 up- and down-regulated genes from each study (S7 Fig). This enrichment score (ES) measures how much the top DEG in one study is over-represented among the top DEG in all the other studies, providing a consistency check for the direction of gene regulation. A positive score means that a gene is often found to be overexpressed in AF across multiple studies. Conversely, a negative score would mean that a gene is often found to be less transcribed in AF samples across these studies. In our results, the median ES for the differentially up-regulated genes in the L- and R-CS were 0.38 and 0.31, respectively (S7C and S7G Figs). On the other hand, the ES values for the down-regulated genes in the L- and R-CS were -0.37 and -0.33, respectively (S7D and S7H Figs).

Moreover, a significant correlation between the AUROC values of the disease score and enrichment scores for DEGs was observed for both the L-CS (Pearson correlations of 0.77 for up-regulated genes [p < 0.001] and -0.67 for down-regulated genes [p < 0.001]) and the R-CS (Pearson correlations of 0.80 for up-regulated genes [p < 0.001] and -0.73 for down-regulated genes [p < 0.001]). This finding suggests that, despite study-specific variations, the direction of gene expression changes was consistent across studies. Additional analyses showed similar results when different numbers of top genes were selected (50, 100, 200, 500, and 1000) (S8 Fig).

### Meta-analysis of transcriptional signatures in AF

After confirming the feasibility of integrating the assessed data, we developed the AF-CS for each atrial chamber by pooling the individual-study results of 21,722 genes for the LAA and 11,436 for the RAA contrasts using the Fisher’s Method (S5 Table). This consensus ranking represents the overall differential gene regulation patterns across the AF spectrum, highlighting genes consistently deregulated in common directions over multiple studies (S9 Fig). In the LAA, 650 genes showed a BH p-value < 0.05; this number was 203 for the RAA analysis (S5 Table). Several genes related to structural cardiac remodeling processes were observed among the top positions of the AF-CS in both cardiac chambers, including COLQ, ANGPTL2, LBH, and COL21A1 [76–79]. Other genes near the top of the consensus included those involved in myocardial electrical remodeling processes, such as CACNA1G, HCN4, and KCNK3 [80–82]. Multiple genes related to the activity of the innate immune system were also found to be significant and highly ranked in the AF-CS, especially in the LAA, with ASAH1, CKAP4, STING1, and FCER1G standing out. Finally, new genes not previously related to AF in the literature were identified as occupying leading positions in the consensus, including DHRS9, CRTAC1, RASL11B, and GRIP2 (S10 Fig).

The leave-one-out sensitivity analysis confirmed the robustness of the overall ranking results, as the resulting gene ranks were consistently preserved after removing one study at a time (mean Spearman rank correlation: LAA = 0.96 and RAA = 0.94) (S11 Fig). There were no significant associations between the sample size of each analyzed study and the enrichment of its specific DEG in the higher positions of the CS (LAA: Spearman correlation, 0.58, P = 0.07; RAA: Spearman correlation, 0.63, P = 0.15). Despite the small sample sizes observed in some studies, this result indicated that most studies were robust enough to provide real biological insights from the contrasts performed.

When analyzing the top 500 genes of each individual chamber’s consensus signature, we observed a low concordance of the genes occupying these positions (Jaccard index value: 0.17). However, a threshold-free approach (RRHO test) of the whole gene rankings revealed a significant overlap in both directions of gene expression between the two chambers, highlighting a robust overall concordance between the two signatures (S12 Fig).

We then assessed the added value of performing a transcriptional consensus meta-analysis in this context by comparing the performance of the disease score constructed using the top 500 meta-analysis genes vs. individual studies. We observed superior predictive performance of the measures derived from the meta-analysis in both LAA (mean AUC AF-CS: 0.82 vs. mean AUC individual studies: 0.74. p < 0.001) and RAA (mean AUC AF-CS: 0.90 vs. mean AUC individual studies: 0.77. p < 0.001). Similarly, the mean enrichment scores obtained from the AF-CS results were significantly better compared to the ones from individual studies for both up-regulated genes (LAA: mean ES 0.52 vs. 0.38. p < 0.001; RAA: mean ES 0.47 vs. 0.30. p < 0.001) and down-regulated genes (LAA: mean ES -0.42 vs. -0.33. p = 0.002; RAA: mean ES -0.50 vs. -0.37. p < 0.001). When testing whether the top meta-analysis genes of each chamber’s AF-CS could predict the disease condition in samples from the other chamber, we observed significantly better performance in terms of the median AUC compared with the pairwise classifier from individual study data (LAA CS in RAA samples: 0.89 vs. individual RAA studies: 0.77. p < 0.001; RAA CS in LAA samples: 0.77 vs. individual LAA studies: 0.74. p < 0.001). These results suggested more consistent biological signals with the meta-analysis results over individual analyses and supported the presence of common gene expression patterns in both chambers.

### Functional analysis of the AF-CS

**Fig 3:**
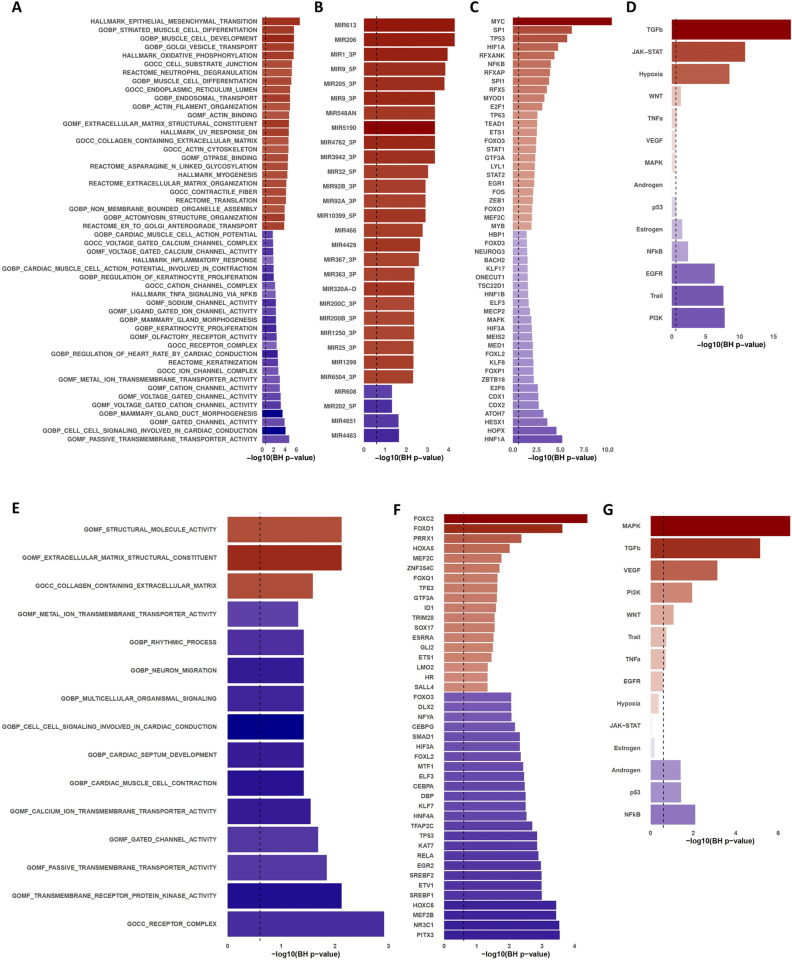
Functional analysis results of the AF-CS in each cardiac chamber. Bar length indicates the statistical significance of the specific item (gene set, miRNA, TF, pathway) enrichment (−log10 BH P‐values) with the vertical dashed line indicating the statistical significance threshold (Benjamini‐Hochberg P = 0.25) and bar color indicating the direction of enrichment (A, B, E, and F) or the direction of activation (C, D, G, and H). Top 25 (A) most enriched canonical, ontology, and hallmark gene sets, along with Reactome and KEGG pathways, (B) miRNAs’ targets, (C) transcription factor activities, and (D) signaling pathway activities in each direction from the L-CS. Top 25 (E) most enriched canonical, ontology, and hallmark gene sets, along with Reactome and KEGG pathways, (F) transcription factor activities, and (G) all signaling pathway activities in each direction from the RAA AF-CS.

The AF-CS results allowed us to characterize the cellular processes consistently deregulated in AF. To this end, we estimated the activity of transcription factors, signaling pathways, and miRNAs in each of the cardiac chambers using a gene set enrichment analysis approach (Fig 3). We found significant enrichment for the LAA consensus signature for 366 Gene Ontology terms, 28 Hallmark gene sets, 42 Reactome pathways, and 25 KEGG pathways (S6 Table). Positively enriched gene sets in this chamber were predominantly related to epithelial-mesenchymal transition, extracellular collagen matrix organization, and cytoskeletal regulation. In contrast, negatively enriched sets were associated with diverse processes related to electrical/muscle function, neuronal signaling, muscle contraction, and ion channel complexes. On the other hand, the number of results in the R-CS enrichment was lower, with only 15 significantly enriched Gene Ontology terms. These included upregulated processes related to extracellular collagen matrix organization; however, most of the enriched terms were observed to be downregulated in the AF group, highlighting cardiac conduction, autonomic function, action potential, and cell-cell signaling processes. When comparing the top results between the chambers, collagen extracellular matrix organization processes were significantly upregulated in both atrial appendages during AF, underlining the role of extracellular matrix remodeling in AF pathophysiology. On the other hand, processes related to normal cardiac conduction and signaling mechanisms were universally downregulated in AF. Notably, we did not find processes that were significantly upregulated in LAA and simultaneously downregulated in RAA, or vice versa, suggesting that differential regulation of processes between atrial appendages, if present, is likely to be nuanced.

Furthermore, the inferred transcriptional activity of 754 transcription factors (TF) allowed us to identify 100 differentially active TF in the LAA and 74 in the RAA. At the same time, 100 miRNA targets were differentially enriched in AF patients in the LAA (S6 Table). Finally, pathway analysis reported the up-regulation of the TGFβ, JAK-STAT, Hypoxia, and WNT pathways in the LAA, with a simultaneous down-regulation of the PI3K, TRAIL, EGFR, NF-kB, and Estrogen pathways in this chamber. In contrast, the RAA results suggested an up-regulation of the TGFβ, MAPK, PI3K and VEGF pathways, while the Androgen, p53 and NF-kB pathways were observed to be downregulated. These results highlighted the divergent transcriptional landscapes and the potential chamber-specific molecular changes that underlie the complexity of atrial fibrillation pathophysiology.

### Tissue-cell-type enrichment of the AF-CS

To get insights into how processes enriched in the AF-CS may relate to the function of individual cell types, we performed a tissue- and cell-type-specific enrichment analysis using WebCSEA (see Methods). At the selected Bonferroni-corrected significance threshold (p = 3.69 x 10^-5), the signatures of two sets of fetal cardiomyocytes were significantly enriched in the L-CS (Fetal_HCA_Heart_Cardiomyocytes: p-value = 8.43 x 10^-6 and FetalHeart_Ventricle cardiomyocyte: p-value = 2.63 x 10^-5), while the signatures of a fibroblast cell-type (AdultArtery_Fibroblast: p-value = 1.61 x 10^-5) and a fetal mesenchymal progenitor (FetalBrain_Fetal mesenchymal progenitor: p-value = 2.67 x 10^-5) were significantly enriched in the RAA CS (S13 Fig). These results highlight the reactivation of fetal cellular programs during the cardiac remodeling process in AF-affected atria.

### Enrichment of GWAS-derived AF genes in the AF-CS

We explored whether the AF consensus signature included genes previously observed to be relevant for AF susceptibility in GWAS studies. To this end, we tested whether the number of AF susceptibility genes by GWAS in the NHGRI-EBI GWAS Catalog was significantly higher in the consensus AF transcriptomic signature of each chamber than would be expected by chance, observing a significant enrichment of AF susceptibility genes in both the LAA (n = 13; p < 0.001) and RAA (n = 8; p = 0.001) consensus signatures [83]. Among the shared genes we highlight the interleukin-6 (IL-6) receptor (IL6R), as single nucleotide variants (SNV) at this gene locus have been associated with AF development and recurrence after catheter ablation and its expression was consistently dysregulated in our AF consensus signatures for both chambers [84–88].

### Performance of the AF-CS as predictors of AF status

We evaluated the predictive potential of the AF-CS derived from atrial myocardium in two distinct contexts: peripheral blood and left ventricular myocardium derived from AF and SR patients in publicly available datasets [89–91]. This approach aimed to assess the broader applicability and potential clinical utility of these signatures.

First, we explored the performance of AF-CS in peripheral blood samples, considering its potential as a non-invasive biomarker for AF. For this purpose, we tested different numbers of top genes from the consensus regarding their performance in sample classification to find the optimal count for each chamber’s signature. As a results, utilizing the top 300 genes from the L-CS and the top 200 genes from the RAA AF-CS, we observed promising discriminatory power between AF and SR patients (AUROC: 0.66–0.75 for LAA; 0.56–0.75 for RAA).

We then extended our analysis to left ventricular myocardium samples from patients with nonischemic (NICM; n = 8) and ischemic (ICM; n = 8) heart failure reported in *Yang et al [*89*]*. f. Interestingly, the AF-CS maintained discriminatory power in this context, suggesting a broader cardiac remodeling process in AF that extends beyond the atria.

These findings highlight the robustness of our AF-CS and its potential translational value, particularly in developing blood-based biomarkers for AF detection and monitoring. Detailed performance metrics for each tissue type and dataset are presented in S7 Table (L-CS) and S8 Table (R-CS), highlighting a significantly better performance of both CS for identifying AF cases from LV samples from the study of *Yang et al* compared to individual studies*.* On the other hand, only the L-CS had a superior performance than individual studies in classifying samples to AF or SR status from peripheral blood samples.

## Discussion

The present transcriptional meta-analysis included 534 samples from 470 patients in 15 studies, representing the largest meta-analysis of transcriptomic data in AF performed to date. Collecting and analyzing this data presented significant challenges, primarily due to the heterogeneity of the original studies in terms of patient populations, sample collection methods, and data processing techniques. Considerable effort was invested in standardizing the data across studies, including rigorous quality control measures, batch effect correction, and the use of a disease scoring metric to enable cross-study gene expression comparability. The robust consensus gene rankings revealed a consistent dysregulation in genes involved in structural and electrical cardiac remodeling, also uncovering novel genes not previously associated with AF. Functional analysis delineated consistently deregulated cellular processes such as epithelial-mesenchymal transition and collagen extracellular matrix organization, while tissue-cell-type enrichment analysis highlighted a significant enrichment of fetal cardiomyocytes in the LAA and fibroblast and fetal mesenchymal progenitor cells in the RAA. Furthermore, we observed a significant enrichment of AF susceptibility genes reported in previous GWAS studies in both consensus signatures. Finally, the AF-CS had a better performance than individual studies in the classification of non-atrial samples according to the AF status. Despite the methodological challenges of meta-analyzing datasets with limited meta-data, our work highlights the value of integrating heterogeneous transcriptomic datasets to identify conserved molecular pathways, thereby contributing to our understanding of AF pathophysiology.

Considering the complex pathophysiological landscape of AF, identifying conserved molecular mechanisms and cellular responses from a transcriptomics perspective represents a significant opportunity to improve our understanding of the phenomena underlying this arrhythmia [92]. Overall, the main processes highlighted in the consensus were related to the structural remodeling of the myocardium [19]. In particular, the functional analysis highlighted the relevance of epithelial-mesenchymal transition (EMT), a widely studied process in HF [93–95]. EMT is characterized by the reactivation of a fetal cellular program involving the expression of embryonic markers in response to myocardial injury [93]. This phenomenon was also observed in the results of the cell type-specific enrichment analysis, suggesting the emergence of cells with a fetal-like phenotype in the AF atria, at least from a genomic perspective. Furthermore, epicardial EMT in response to injury is also associated with increased fibroblast activation and their transformation into myofibroblasts under pathological stress, a finding consistent with the observed enrichment of extracellular matrix-related processes and the fibroblast cell type [93]. The resulting structural alterations and fibrosis development leads to conduction disorders and the reentry phenomena characteristic of AF [92].

Electrophysiological remodeling and calcium (Ca^2+^) handling anomalies were also consistently observed in AF tissues, underlining significant dysregulation of key potassium and calcium channels. These findings were reflected in the significant enrichment of processes pertaining to the action potential, voltage-gated channel activity, and cardiac conduction, all of which were among the top downregulated pathways in both atria. Prior studies have characterized the electrical remodeling present in AF, highlighting the relevance of action potential duration (APD) heterogeneity in the initiation and persistence of reentrant arrhythmias in AF [96,97]. Concurrently, Ca^2+^-handling abnormalities, highlighting RyR2 dysfunction, altered L-type Ca^2+^ currents, and increased spontaneous Ca^2+^ release from the sarcoplasmic reticulum have been implicated as key drivers of AF [98]. Our results are in line with these findings, revealing a conserved dysregulation in critical calcium channels and associated proteins (S100A8, S100A9, SMOC-2) in AF atria, which may support the synergistic role of abnormal Ca^2+^ signaling and structural remodeling in failing cardiomyocyte adaptation to abnormal calcium influx and overload derived from rapid atrial stimulation, thereby facilitating the arrhythmia onset and perpetuation [99].

Furthermore, our findings suggest the presence of metabolic reprogramming processes in AF. These are closely related to EMT, as studies performed in failing hearts have shown that the fetal phenotype is characterized by a “switch back” to glycolysis as the primary energy source of the cardiomyocytes [100]. However, this change may not be straightforward, as the results of our functional analysis suggested an increase in both the activity of oxidative phosphorylation and glycolysis pathways. This apparently discordant finding is consistent with the results of two proteomic studies that reported the upregulation of proteins involved in fatty acid uptake into the mitochondria and β-oxidation, such as fatty acid translocase (FAT/CD36), (14)C-palmitic acid and CD36, which resulted in an apparent increased activity of the oxidative phosphorylation pathway in the AF groups [101,102]. Conversely, they also demonstrated decreased expression and activity in proteins pivotal to oxidative phosphorylation, the TCA cycle, and, notably, the electron transport chain [101,102]. This dichotomy underlines a potentially maladaptive metabolic response to the chronic energy stress inherent in AF, emphasizing that cellular efforts to augment energy production via selective protein upregulation are insufficient to offset the mitochondrial derangements in ATP synthesis [103,104].

The present consensus signature analysis also provided relevant insights regarding the role of the innate immune system in AF, highlighting neutrophil degranulation as the top-ranking Reactome pathway in the L-CS. This finding is supported by current evidence published in the literature, which highlights increased neutrophil extravasation in the atria of AF patients, in addition to observing significant colocalization between enzymes such as myeloperoxidase and areas of atrial myocardial fibrosis [105–107]. On the other hand, the neutrophil-lymphocyte ratio has been shown to perform favorably in predicting de novo AF, as well as relevant clinical outcomes such as stroke [108–110]. Our results extend these findings across the spectrum of AF, highlighting the activity of the innate immune system as a preserved process relevant in the development and persistence of this arrhythmia.

### Strengths and limitations

As a meta-analysis with a standard bioinformatics analysis process, our study was strengthened by the increased sample size compared to individual studies, as well as the feasibility of pooling heterogeneous data, taking into account relevant factors such as the sampling/technical variability and variability derived from significant demographic and clinical covariates. Furthermore, our disease scoring metric enabled cross-study gene expression comparability. Significant directional consistency across studies supported the robustness of our differential gene regulation findings. Notably, including both atrial chambers in our meta-analytic framework provided a granular, chamber-specific perspective on AF pathophysiology, while comprehensive functional analyses yielded actionable insights into the implicated molecular processes. Finally, the validation of the consensus performance in classifying AF cases from other types of samples, specially blood samples, highlights not only the consistency of the signal across tissues and sources, but the potential of transcriptomics-derived markers for future applications in the clinical setting.

Notwithstanding its strengths, our study is subject to several limitations. First, the lack of individual patient data in most incorporated studies limited the possibility for subgroup analysis, and precluded optimal covariate adjustment in multivariate models. Additionally, the studies that met our inclusion criteria included predominantly patients with persistent or permanent AF. Moreover, the low proportion of dual-chamber samples and disparities in gene coverage and sample size between the LAA and RAA impede direct chamber-to-chamber comparability. Another limitation relates to the meta-analysis process itself, as our gene standardization approach could only partially eliminate technical heterogeneity arising from using varied platforms.

### Implications for clinical practice and future directions

The consensus transcriptional signatures derived from this meta-analysis, which consolidates the available knowledge from genome-wide transcriptomics, offer an opportunity to refine the current clinical approach to AF. Identifying consistently dysregulated genes and pathways across heterogeneous patient samples may represent the first step towards identifying and validating novel targeted diagnostic/prognostic biomarkers and provides a molecular framework for designing mechanism-based treatment strategies.

Despite these advances, it is paramount to address the current study’s limitations to foster future research directions. The scarcity of individual patient-level data necessitates further studies to confirm these findings in diverse patient populations, focusing on subgroup analyses to determine the potential effect of concomitant comorbid conditions and to characterize the conserved gene expression patterns in AF across the different types (paroxysmal, persistent, and permanent) to better understand the mechanisms potentially associated with arrhythmia progression and perpetuation. Finally, while we believe our study marks a significant step forward in our understanding of AF, it also delineates the critical avenues for future research aiming to translate these molecular insights into actionable clinical interventions. The stable reference provided by our consensus signatures can serve as a foundation for developing and validating targeted therapies, as well as for identifying novel biomarkers for early detection and risk stratification in AF. This underscores the importance of our work not only in advancing our understanding of AF pathophysiology but also in paving the way for more personalized and effective management strategies for this complex arrhythmia.

### Key messages

*Clinical Relevance for Patient Care:*
*Our meta-analysis of 534 atrial tissue samples provides a comprehensive molecular characterization of AF, revealing consistent biological changes across diverse patient populations.*
***Key Pathological Mechanisms Disrupted in AF:***
*Structural Remodeling*

- Extensive extracellular matrix changes occur in both atria
- Significant activation of fibroblasts and enhanced fibrosis
- Reactivation of fetal gene programs suggests maladaptive cardiac remodeling

*Electrical Remodeling*

- Widespread dysregulation of ion channels and calcium handling proteins
- Altered expression of genes controlling cardiac conduction
- Changes in both action potential and cellular signaling pathways

*Metabolic Remodeling*

- Evidence of altered energy metabolism in AF cardiomyocytes
- Simultaneous upregulation of both glycolysis and oxidative phosphorylation
- Suggests inefficient/insufficient energy production in AF tissue

*Inflammatory Response*

- Activation of innate immune responses, particularly neutrophil-related pathways
- Supports the role of inflammation in AF pathogenesis
- Provides molecular basis for observed elevated inflammatory markers in AF patients

***Diagnostic Applications:***

- The AF molecular signature identified AF patients using blood samples with modest success (AUROC: 0.66–0.75)
- Proof of principle of the potential of blood transcriptomics in AF patient assessment.

## Supporting information

S1 Text Supplemental Text 1. Search strategies used in each evaluated database.
(DOCX)

S1 Table Supplemental Table 1. PRISMA Checklist. (DOCX)

S2 Table Supplemental Table 2. Summary of studies identified in the literature search that were assessed in full-text review. (XLSX)

S3 Table Supplemental Table 3. General characteristics of the datasets included in the meta-analysis. (XLSX)

S4 Table Supplemental Table 4. Methodological quality of included studies using an adapted version of the Checklist for Analytical Cross-Sectional Studies from the Joanna Briggs Institute. (XLSX)

S5 Table Supplemental Table 5. Gene rankings for the LAA and RAA AF-CS. (XLSX)

S6 Table Supplemental Table 6. Results of the functional analysis of the AF-CS in each atrium. (XLSX)

S7 Table Supplemental Table 7. Performance of the top 300 genes from the LAA AF-CS in classifying AF status in non-atrial samples. (XLSX)

S8 Table Supplemental Table 8. Performance of the top 200 genes from the RAA AF-CS in classifying AF status in non-atrial samples. (XLSX)

S1 Fig Supplemental Figure 1. Summary of the analysis process of the atrial fibrillation consensus signature meta-analysis. (DOCX)

S2 Fig Supplemental Figure 2. Age, sex, and left ventricular ejection fraction (LVEF) distribution in the included studies. A) Mean age and standard deviation of the age (years) in atrial fibrillation (AF) and sinus rhythm (SR) groups per study. B) Relative frequencies of each sex per study. C) Mean age and standard deviation of the LVEF (%) in AF and SR groups per study.
(DOCX)

S3 Fig Supplemental Figure 3. Gene coverage summary and comparisons in the included studies. A) Absolute gene coverage per study in the LAA-AF-CS. B) Pairwise comparison of covered genes in the LAA-AF-CS analysis using the Jaccard Index. C) Absolute gene coverage per study in the RAA-AF-CS. D) Pairwise comparison of covered genes in the RAA-CS analysis using the Jaccard Index.
(DOCX)

S4 Fig Supplemental Figure 4. Principal Component Analysis (PCA) of each individual sample in the included studies. A) PCA showing the first two components of the samples from the included studies in the LAA-AF-CS. B) PCA showing the first two components of the AF samples from the included studies in the LAA-AF-CS after z-transforming. C) t-distributed stochastic neighbor embedding (t-SNE) applied to standardized AF samples in the LAA-AF-CS. D) PCA showing the first two components of the samples from the included studies in the RAA-AF-CS. E) PCA showing the first two components of the AF samples from the included studies in the RAA-AF-CS after z-transforming. F) t-distributed stochastic neighbor embedding (t-SNE) applied to standardized AF samples in the RAA-AF-CS.
(DOCX)

S5 Fig Supplemental Figure 5. PCA showing the first two components after gene standardization and the cumulative proportion of variance explained by components related to AF, study, technology used, age, sex, and LVEF. A) LAA-AF-CS. B) RAA-AF-CS.
(DOCX)

S6 Fig Supplemental Figure 6. Summary of distributions of -log10(p-values), t-values, and log2(fold-changes) derived from the differential expression analyses. A) LAA-AF-CS. B) RAA-AF-CS.
(DOCX)

S7 Fig Supplemental Figure 7. Consistency of the transcriptomic signatures of AF across the included studies. (DOCX)

S8 Fig Supplemental Figure 8. Evaluation of the reliability of measures for comparing study results in the meta-analysis. A) Disease Score in the LAA-AF-CS. B) Enrichment Score in the LAA-AF-CS. C) Disease Score in the RAA-AF-CS. D) Enrichment Score in the RAA-AF-CS.
(DOCX)

S9 Fig Supplemental Figure 9. Sorted meta-analysis BH -log10(p-values) derived from the Fisher combined test. A) LAA-AF-CS. B) RAA-AF-CS.
(DOCX)

S10 Fig Supplemental Figure 10. Summary of the top-ranking genes according to log-fold change (LFC) and position in the consensus signature. A) LAA-AF-CS. B) RAA-AF-CS.
(DOCX)

S11 Fig Supplemental Figure 11. Results of leave-one-out sensitivity analysis. A) LAA-AF-CS. B) RAA-AF-CS.
(DOCX)

S12 Fig Supplemental Figure 12. Rank Rank Hypergeometric Overlap (RRHO) heatmap comparing the gene expression signatures from the LAA-AF-CS and RAA-AF-CS. (DOCX)

S13 Fig Supplemental Figure 13. Tissue-cell-type enrichment analysis derived from the consensus signature rankings. A) LAA-AF-CS. B) RAA-AF-CS.
(DOCX)

## References

[1] Lippi G, Sanchis-Gomar F, Cervellin G. Global epidemiology of atrial fibrillation: An increasing epidemic and public health challenge. Int J Stroke. 2021;16(2):217–21. doi: 10.1177/1747493019897870 31955707

[2] Hindricks G, Potpara T, Dagres N, Arbelo E, Bax JJ, Blomström-Lundqvist C, et al. 2020 ESC guidelines for the diagnosis and management of atrial fibrillation developed in collaboration with the European Association for Cardio-Thoracic Surgery (EACTS): The Task Force for the diagnosis and management of atrial fibrillation of the European Society of Cardiology (ESC) Developed with the special contribution of the European Heart Rhythm Association (EHRA) of the ESC. Eur Heart J. 2021;42(5):373–498. doi: 10.1093/eurheartj/ehaa612 32860505

[3] Delaney JA, Yin X, Fontes JD, Wallace ER, Skinner A, Wang N, et al. Hospital and clinical care costs associated with atrial fibrillation for Medicare beneficiaries in the Cardiovascular Health Study and the Framingham Heart Study. SAGE Open Med. 2018;6:2050312118759444. doi: 10.1177/2050312118759444 29511541 PMC5826000

[4] Farmakis D, Chrysohoou C, Giamouzis G, Giannakoulas G, Hamilos M, Naka K, et al. The management of atrial fibrillation in heart failure: An expert panel consensus. Heart Fail Rev. 2021;26(6):1345–58. doi: 10.1007/s10741-020-09978-0 32468277

[5] Kraft M, Büscher A, Wiedmann F, L’hoste Y, Haefeli WE, Frey N, et al. Current drug treatment strategies for atrial fibrillation and TASK-1 inhibition as an emerging novel therapy option. Front Pharmacol. 2021;12:638445. doi: 10.3389/fphar.2021.638445 33897427 PMC8058608

[6] Reiffel JA. Selected advancements in the management of atrial fibrillation from the year 2021. J Innov Card Rhythm Manag. 2022;13(1):4840–6. doi: 10.19102/icrm.2022.130107 35127237 PMC8812470

[7] Sohinki D, Stavrakis S. New approaches for treating atrial fibrillation: Focus on autonomic modulation. Trends Cardiovasc Med. 2020;30(7):433–9. doi: 10.1016/j.tcm.2019.10.009 31708408 PMC7190441

[8] Chung M, Youn S, Barnard J. Insights from atrial fibrillation genomics: From bedside to bench and back again. Cardiol Rev. 2019;27:302–7. 10.1097/CRD.0000000000000267 31584471 10.1097/CRD.0000000000000267 PMC8421131

[9] Lin H, Yin X, Lunetta KL, Dupuis J, McManus DD, Lubitz SA, et al. Whole blood gene expression and atrial fibrillation: The Framingham Heart Study. PLoS One. 2014;9(5):e96794. doi: 10.1371/journal.pone.0096794 24805109 PMC4013062

[10] van Rooij E, Sutherland LB, Liu N, Williams AH, McAnally J, Gerard RD, et al. A signature pattern of stress-responsive microRNAs that can evoke cardiac hypertrophy and heart failure. Proc Natl Acad Sci U S A. 2006;103(48):18255–60. doi: 10.1073/pnas.0608791103 17108080 PMC1838739

[11] Dawson K, Wakili R, Ordög B, Clauss S, Chen Y, Iwasaki Y, et al. MicroRNA29: a mechanistic contributor and potential biomarker in atrial fibrillation. Circulation. 2013;127(14):1466–75, 1475e1-28. doi: 10.1161/CIRCULATIONAHA.112.001207 23459615

[12] Cong H, Li X, Ma L, Jiang H, Mao Y, Xu M. Angiotensin II receptor type 1 is upregulated in atrial tissue of patients with rheumatic valvular disease with atrial fibrillation. J Thorac Cardiovasc Surg. 2010;140(2):298–304. doi: 10.1016/j.jtcvs.2009.10.035 20080265

[13] Jiang Y-Y, Hou H-T, Yang Q, Liu X-C, He G-W. Chloride channels are involved in the development of atrial fibrillation – A transcriptomic and proteomic study. Sci Rep. 2017;7(1):10215. doi: 10.1038/s41598-017-10590-w 28860555 PMC5579191

[14] Steenman M. Insight into atrial fibrillation through analysis of the coding transcriptome in humans. Biophys Rev. 2020;12(4):817–26. doi: 10.1007/s12551-020-00735-z 32666467 PMC7429641

[15] Shoemaker MB, Roden DM. Atrial Fibrillation Is a Complex Trait: Very Complex. Circ Res. 2020;127(2):244–6. doi: 10.1161/CIRCRESAHA.120.317112 32614720 PMC7337979

[16] Tucker NR, Clauss S, Ellinor PT. Common variation in atrial fibrillation: navigating the path from genetic association to mechanism. Cardiovasc Res. 2016;109(4):493–501. doi: 10.1093/cvr/cvv283 26733238 PMC4777911

[17] Sung J, Wang Y, Chandrasekaran S, Witten DM, Price ND. Molecular signatures from omics data: From chaos to consensus. Biotechnol J. 2012;7(8):946–57. doi: 10.1002/biot.201100305 22528809 PMC3418428

[18] Tajti F, Kuppe C, Antoranz A, Ibrahim MM, Kim H, Ceccarelli F, et al. A functional landscape of CKD entities from public transcriptomic data. Kidney Int Rep. 2019;5(2):211–24. doi: 10.1016/j.ekir.2019.11.005 32043035 PMC7000845

[19] Ramirez Flores RO, Lanzer JD, Holland CH, Leuschner F, Most P, Schultz J-H, et al. Consensus transcriptional landscape of human end-stage heart failure. J Am Heart Assoc. 2021;10(7):e019667. doi: 10.1161/JAHA.120.019667 33787284 PMC8174362

[20] Page MJ, McKenzie JE, Bossuyt PM, Boutron I, Hoffmann TC, Mulrow CD, et al. The PRISMA 2020 statement: An updated guideline for reporting systematic reviews. BMJ. 2021;372:n71. doi: 10.1136/bmj.n71 33782057 PMC8005924

[21] Glisic M, Raguindin PF, Gemperli A, Taneri PE, Salvador D Jr, Voortman T, et al. A 7-step guideline for qualitative synthesis and meta-analysis of observational studies in health Sciences. Public Health Rev. 2023;44:1605454. 10.3389/phrs.2023.1605454 37260612 PMC10227668

[22] Muka T, Glisic M, Milic J, Verhoog S, Bohlius J, Bramer W, et al. A 24-step guide on how to design, conduct, and successfully publish a systematic review and meta-analysis in medical research. Eur J Epidemiol. 2020;35(1):49–60. doi: 10.1007/s10654-019-00576-5 31720912

[23] Benjamini Y, Hochberg Y. Controlling the false discovery rate: a practical and powerful approach to multiple testing. J R Stat Soc. 1995;57:289–300. 10.1111/j.2517-6161.1995.tb02031.x

[24] Plaisier SB, Taschereau R, Wong JA, Graeber TG. Rank-rank hypergeometric overlap: Identification of statistically significant overlap between gene-expression signatures. Nucleic Acids Res. 2010;38(17):e169. doi: 10.1093/nar/gkq636 20660011 PMC2943622

[25] Müller-Dott S, Tsirvouli E, Vazquez M, Ramirez Flores RO, Badia-I-Mompel P, Fallegger R, et al. Expanding the coverage of regulons from high-confidence prior knowledge for accurate estimation of transcription factor activities. Nucleic Acids Res. 2023;51(20):10934–49. doi: 10.1093/nar/gkad841 37843125 PMC10639077

[26] Badia-I-Mompel P, Vélez Santiago J, Braunger J, Geiss C, Dimitrov D, Müller-Dott S. DecoupleR: ensemble of computational methods to infer biological activities from omics data. Bioinform Adv. 2022;2:vbac016. doi: 10.1093/bioadv/vbac016 36699385 PMC9710656

[27] Garcia-Alonso L, Holland CH, Ibrahim MM, Turei D, Saez-Rodriguez J. Benchmark and integration of resources for the estimation of human transcription factor activities. Genome Res. 2019;29(8):1363–75. doi: 10.1101/gr.240663.118 31340985 PMC6673718

[28] Schubert M, Klinger B, Klünemann M, Sieber A, Uhlitz F, Sauer S, et al. Perturbation-response genes reveal signaling footprints in cancer gene expression. Nat Commun. 2018;9(1):20. doi: 10.1038/s41467-017-02391-6 29295995 PMC5750219

[29] Adam O, Lavall D, Theobald K, Hohl M, Grube M, Ameling S, et al. Rac1-induced connective tissue growth factor regulates connexin 43 and N-cadherin expression in atrial fibrillation. J Am Coll Cardiol. 2010;55(5):469–80. doi: 10.1016/j.jacc.2009.08.064 20117462

[30] Assum I, Krause J, Scheinhardt M, Müller C, Hammer E, Börschel C. Tissue-specific multi-omics analysis of atrial fibrillation. Nat Commun. 2022;13:441. doi: 10.1038/s41467-022-27953-1 35064145 PMC8782899

[31] Barth AS, Merk S, Arnoldi E, Zwermann L, Kloos P, Gebauer M, et al. Reprogramming of the human atrial transcriptome in permanent atrial fibrillation: Expression of a ventricular-like genomic signature. Circ Res. 2005;96(9):1022–9. doi: 10.1161/01.RES.0000165480.82737.33 15817885

[32] Chen H-X, Li M-Y, Jiang Y-Y, Hou H-T, Wang J, Liu X-C, et al. Role of the PPAR pathway in atrial fibrillation associated with heart valve disease: Transcriptomics and proteomics in human atrial tissue. Signal Transduct Target Ther. 2020;5(1):4. doi: 10.1038/s41392-019-0093-2 32296022 PMC6971265

[33] Chiang DY, Zhang M, Voigt N, Alsina KM, Jakob H, Martin JF, et al. Identification of microRNA-mRNA dysregulations in paroxysmal atrial fibrillation. Int J Cardiol. 2015;184:190–7. doi: 10.1016/j.ijcard.2015.01.075 25706326 PMC4417399

[34] Darkow E, Nguyen TT, Stolina M, Kari FA, Schmidt C, Wiedmann F, et al. Small conductance Ca2 +-activated K+ (SK) channel mRNA expression in human atrial and ventricular tissue: Comparison between donor, atrial fibrillation and heart failure tissue. Front Physiol. 2021;12:650964. doi: 10.3389/fphys.2021.650964 33868017 PMC8047327

[35] Çubukçuoğlu Deniz G, Durdu S, Doğan Y, Erdemli E, Özdağ H, Akar AR. Molecular signatures of human chronic atrial fibrillation in primary mitral regurgitation. Cardiovasc Ther. 2021;2021:5516185. doi: 10.1155/2021/5516185 34737791 PMC8538404

[36] Deshmukh A, Barnard J, Sun H, Newton D, Castel L, Pettersson G, et al. Left atrial transcriptional changes associated with atrial fibrillation susceptibility and persistence. Circ Arrhythm Electrophysiol. 2015;8(1):32–41. doi: 10.1161/CIRCEP.114.001632 25523945 PMC4334691

[37] DoñatePuertas R, Meugnier E, Romestaing C, Rey C, Morel E, Lachuer J. Atrial fibrillation is associated with hypermethylation in human left atrium, and treatment with decitabine reduces atrial tachyarrhythmias in spontaneously hypertensive rats. Transl Res. 2017;184:57-67.e5. doi: 10.1016/j.trsl.2017.03.004 28427903

[38] Herrera-Rivero M, Gandhi S, Witten A, Ghalawinji A, Schotten U, Stoll M. Cardiac chamber-specific genetic alterations suggest candidate genes and pathways implicating the left ventricle in the pathogenesis of atrial fibrillation. Genomics. 2022;114(2):110320. doi: 10.1016/j.ygeno.2022.110320 35218871

[39] Hsu J, Gore-Panter S, Tchou G, Castel L, Lovano B, Moravec CS, et al. Genetic control of left atrial gene expression yields insights into the genetic susceptibility for atrial fibrillation. Circ Genom Precis Med. 2018;11(3):e002107. doi: 10.1161/CIRCGEN.118.002107 29545482 PMC5858469

[40] Kertai MD, Qi W, Li Y-J, Lombard FW, Liu Y, Smith MP, et al. Gene signatures of postoperative atrial fibrillation in atrial tissue after coronary artery bypass grafting surgery in patients receiving β-blockers. J Mol Cell Cardiol. 2016;92:109–15. doi: 10.1016/j.yjmcc.2016.02.006 26860460 PMC4967350

[41] Kharlap MS, Timofeeva AV, Goryunova LE, Khaspekov GL, Dzemeshkevich SL, Ruskin VV, et al. Atrial appendage transcriptional profile in patients with atrial fibrillation with structural heart diseases. Ann N Y Acad Sci. 2006;1091:205–17. doi: 10.1196/annals.1378.067 17341615

[42] Kim N-H, Ahn Y, Oh SK, Cho JK, Park HW, Kim Y-S, et al. Altered patterns of gene expression in response to chronic atrial fibrillation. Int Heart J. 2005;46(3):383–95. doi: 10.1536/ihj.46.383 16043935

[43] Kim YH, Lim DS, Lee JH, Shim WJ, Ro YM, Park GH, et al. Gene expression profiling of oxidative stress on atrial fibrillation in humans. Exp Mol Med. 2003;35(5):336–49. doi: 10.1038/emm.2003.45 14646586

[44] Lal JC, Mao C, Zhou Y, Gore-Panter SR, Rennison JH, Lovano BS, et al. Transcriptomics-based network medicine approach identifies metformin as a repurposable drug for atrial fibrillation. Cell Rep Med. 2022;3(10):100749. doi: 10.1016/j.xcrm.2022.100749 36223777 PMC9588904

[45] Lamirault G, Gaborit N, Le Meur N, Chevalier C, Lande G, Demolombe S, et al. Gene expression profile associated with chronic atrial fibrillation and underlying valvular heart disease in man. J Mol Cell Cardiol. 2006;40(1):173–84. doi: 10.1016/j.yjmcc.2005.09.004 16242148

[46] Lipovsky CE, Jimenez J, Guo Q, Li G, Yin T, Hicks SC, et al. Chamber-specific transcriptional responses in atrial fibrillation. JCI Insight. 2020;5(18):e135319. doi: 10.1172/jci.insight.135319 32841220 PMC7526559

[47] Mei B, Liu H, Yang S, Liang M-Y, Yue Y, Huang S-Q, et al. Long non-coding RNA expression profile in permanent atrial fibrillation patients with rheumatic heart disease. Eur Rev Med Pharmacol Sci. 2018;22(20):6940–7. doi: 10.26355/eurrev_201810_16165 30402860

[48] Ohki R, Yamamoto K, Ueno S, Mano H, Misawa Y, Fuse K, et al. Gene expression profiling of human atrial myocardium with atrial fibrillation by DNA microarray analysis. Int J Cardiol. 2005;102(2):233–8. doi: 10.1016/j.ijcard.2004.05.026 15982490

[49] Larupa Santos J, Rodríguez I, S Olesen M, Hjorth Bentzen B, Schmitt N. Investigating gene-microRNA networks in atrial fibrillation patients with mitral valve regurgitation. PLoS One. 2020;15(5):e0232719. doi: 10.1371/journal.pone.0232719 32392228 PMC7213724

[50] Sun H, Shao Y. Transcriptome analysis reveals key pathways that vary in patients with paroxysmal and persistent atrial fibrillation. Exp Ther Med. 2021;21:1–13. 33850543 10.3892/etm.2021.10003 PMC8027719

[51] Thomas AM, Cabrera CP, Finlay M, Lall K, Nobles M, Schilling RJ, et al. Differentially expressed genes for atrial fibrillation identified by RNA sequencing from paired human left and right atrial appendages. Physiol Genomics. 2019;51(8):323–32. doi: 10.1152/physiolgenomics.00012.2019 31172864 PMC6732415

[52] Tsai F-C, Lin Y-C, Chang S-H, Chang G-J, Hsu Y-J, Lin Y-M, et al. Differential left-to-right atria gene expression ratio in human sinus rhythm and atrial fibrillation: Implications for arrhythmogenesis and thrombogenesis. Int J Cardiol. 2016;222:104–12. doi: 10.1016/j.ijcard.2016.07.103 27494721

[53] van den Berg NWE, Kawasaki M, Fabrizi B, Nariswari FA, Verduijn AC, Neefs J, et al. Epicardial and endothelial cell activation concurs with extracellular matrix remodeling in atrial fibrillation. Clin Transl Med. 2021;11(11):e558. doi: 10.1002/ctm2.558 34841686 PMC8567047

[54] Wang R, Bektik E, Sakon P, Wang X, Huang S, Meng X, et al. Integrated analysis of the microRNA-mRNA network predicts potential regulators of atrial fibrillation in humans. Cells. 2022;11(17):2629. doi: 10.3390/cells11172629 36078037 PMC9454849

[55] Wu N, Li J, Chen X, Xiang Y, Wu L, Li C, et al. Identification of long non-coding RNA and circular RNA expression profiles in atrial fibrillation. Heart Lung Circ. 2020;29(7):e157–67. doi: 10.1016/j.hlc.2019.10.018 31843366

[56] Yeh Y-H, Kuo C-T, Lee Y-S, Lin Y-M, Nattel S, Tsai F-C, et al. Region-specific gene expression profiles in the left atria of patients with valvular atrial fibrillation. Heart Rhythm. 2013;10(3):383–91. doi: 10.1016/j.hrthm.2012.11.013 23183193

[57] Zeemering S, Isaacs A, Winters J, Maesen B, Bidar E, Dimopoulou C, et al. Atrial fibrillation in the presence and absence of heart failure enhances expression of genes involved in cardiomyocyte structure, conduction properties, fibrosis, inflammation, and endothelial dysfunction. Heart Rhythm. 2022;19(12):2115–24. doi: 10.1016/j.hrthm.2022.08.019 36007727

[58] Zhang Y, Ke X, Liu J, Ma X, Liu Y, Liang D, et al. Characterization of circRNA‑associated ceRNA networks in patients with nonvalvular persistent atrial fibrillation. Mol Med Rep. 2019;19(1):638–50. doi: 10.3892/mmr.2018.9695 30483740

[59] Zhang Y, Shen H, Wang P, Min J, Yu Y, Wang Q, et al. Identification and characterization of circular RNAs in atrial appendage of patients with atrial fibrillation. Exp Cell Res. 2020;389(2):111821. doi: 10.1016/j.yexcr.2020.111821 31923425

[60] Zhou J, Gao J, Liu Y, Gu S, Zhang X, An X, et al. Human atrium transcript analysis of permanent atrial fibrillation. Int Heart J. 2014;55(1):71–7. doi: 10.1536/ihj.13-196 24463922

[61] Zhu X, Tang X, Chong H, Cao H, Fan F, Pan J, et al. Expression profiles of circular RNA in human atrial fibrillation with valvular heart diseases. Front Cardiovasc Med. 2020;7:597932. doi: 10.3389/fcvm.2020.597932 33330659 PMC7714832

[62] Igarashi W, Takagi D, Okada D, Kobayashi D, Oka M, Io T, et al. Bioinformatic Identification of Potential RNA Alterations on the Atrial Fibrillation Remodeling from Human Pulmonary Veins. Int J Mol Sci. 2023;24(13):10501. doi: 10.3390/ijms241310501 37445678 PMC10342093

[63] Adam O, Lavall D, Theobald K, Hohl M, Grube M, Ameling S, et al. GSE14975 [Internet]. Rac1-Induced Connective Tissue Growth Factor regulates Connexin 43 and N-Cadherin Expression in Atrial Fibrillation. 2009 [cited 2023 Nov 6]. Available from: https://www.ncbi.nlm.nih.gov/geo/query/acc.cgi?acc=GSE14975 10.1016/j.jacc.2009.08.064 20117462

[64] Barth AS, Merk S, Arnoldi E, Zwermann L, Kloos P, Gebauer M, et al. GSE2240 [Internet]. Reprogramming of the human atrial transcriptome in permanent atrial fibrillation: expression of a ventricular-like genomic signature. 2005 [cited 2023 Nov 6]. Available from: https://www.ncbi.nlm.nih.gov/geo/query/acc.cgi?acc=GSE2240 10.1161/01.RES.0000165480.82737.33 15817885

[65] Çubukçuoğlu Deniz G, Durdu S, Doğan Y, Erdemli E, et al. GSE115574 [Internet]. Molecular Signatures of Human Chronic Atrial Fibrillation in Primary Mitral Regurgitation. 2018 [cited 2023 Nov 6]. Available from: https://www.ncbi.nlm.nih.gov/geo/query/acc.cgi?acc=GSE115574 10.1155/2021/5516185 PMC8538404 34737791

[66] Hsu J, Gore-Panter S, Tchou G, Castel L, Lovano B, Moravec CS, et al. GSE69890 [Internet]. The Genetic Control of Left Atrial Gene Expression Yields Insights into the Genetic Susceptibility for Atrial Fibrillation. 2015 [cited 2023 Nov 6]. Available from: https://www.ncbi.nlm.nih.gov/geo/query/acc.cgi?acc=GSE69890 10.1161/CIRCGEN.118.002107 PMC5858469 29545482

[67] Morel E, Chevalier P, Rome S, Meugnier E. GSE31821 [Internet]. Gene expression and miRNA profiles in atrial fibrillation. Role of miR-21. 2011 [cited 2023 Nov 6]. Available from: https://www.ncbi.nlm.nih.gov/geo/query/acc.cgi?acc=GSE31821

[68] Thomas AM, Cabrera CP, Finlay M, Lall K, Nobles M, Schilling RJ, et al. GSE128188 [Internet]. Differentially expressed genes for atrial fibrillation identified by RNA sequencing from paired human left and right atrial appendages. 2019 [cited 2023 Nov 6]. Available from: https://www.ncbi.nlm.nih.gov/geo/query/acc.cgi?acc=GSE128188 10.1152/physiolgenomics.00012.2019 PMC6732415 31172864

[69] Tsai F, Li Y, Chang S, Chang G, Hsu Y, Lin Y, et al. GSE79768 [Internet]. Differential left-to-right atria gene expression ratio in human sinus rhythm and atrial fibrillation: Implications for arrhythmogenesis and thrombogenesis. 2016 [cited 2023 Nov 6]. Available from: https://www.ncbi.nlm.nih.gov/geo/query/acc.cgi?acc=GSE79768 10.1016/j.ijcard.2016.07.103 27494721

[70] Yeh Y, Kou C, Chan Y, Lin Y, Lee Y, Lin Y, et al. GSE41177 [Internet]. Region-specific gene expression profiles in the left atria of patients with valvular atrial fibrillation. 2012 [cited 2023 Nov 6]. Available from: https://www.ncbi.nlm.nih.gov/geo/query/acc.cgi?acc=GSE41177 10.1016/j.hrthm.2012.11.013 23183193

[71] Darkow E, Nguyen TT, Stolina M, Kari FA, Schmidt C, Wiedmann F, et al. PRJEB42485 [Internet]. Small Conductance Ca2 +-Activated K+ (SK) Channel mRNA Expression in Human Atrial and Ventricular Tissue: Comparison Between Donor, Atrial Fibrillation and Heart Failure Tissue. 2021 [cited 2023 Nov 6]. Available from: https://www.ebi.ac.uk/ena/browser/view/PRJEB42485 10.3389/fphys.2021.650964 PMC8047327 33868017

[72] Sun HY. PRJNA531935 [Internet]. Transcriptome analysis reveals key pathways that vary in patients with paroxysmal and persistent atrial fibrillation. 2019. Available from: https://www.ebi.ac.uk/ena/browser/view/PRJNA531935 10.3892/etm.2021.10003 PMC8027719 33850543

[73] Herrera-Rivero M, Gandhi S, Witten A, Ghalawinji A, Schotten U, Stoll M, et al. PRJNA771631 [Internet]. Cardiac chamber-specific genetic alterations suggest candidate genes and pathways implicating the left ventricle in the pathogenesis of atrial fibrillation. 2022. Available from: https://www.ebi.ac.uk/ena/browser/view/PRJNA771631?show=parent-projects 10.1016/j.ygeno.2022.110320 35218871

[74] Zhu X, Tang X, Chong H, Cao H, Fan F, Pan J, et al. PRJNA667522 [Internet]. Expression Profiles of Circular RNA in Human Atrial Fibrillation With Valvular Heart Diseases. 2020. Available from: https://www.ebi.ac.uk/ena/browser/view/PRJNA667522 10.3389/fcvm.2020.597932 PMC7714832 33330659

[75] Larupa Santos J, Rodríguez IS, Olesen M, Hjorth Bentzen B, Schmitt N. RNA_seq_human_RA [Internet]. Investigating gene-microRNA networks in atrial fibrillation patients with mitral valve regurgitation. 2019. Available from: https://erda.ku.dk/public/archives/41ca475d27ef9f23a58ed07fecbdc046/published-archive.html 10.1371/journal.pone.0232719 PMC7213724 32392228

[76] Xu Y, Wu A, Chen J, Song X, Chen M, Liu Q. Limb-Bud and Heart (LBH) upregulation in cardiomyocytes under hypoxia promotes the activation of cardiac fibroblasts via exosome secretion. Mediators Inflamm. 2022;2022:8939449. doi: 10.1155/2022/8939449 36110098 PMC9470350

[77] Gil-Cayuela C, Roselló-LLetí E, Ortega A, Tarazón E, Triviño JC, Martínez-Dolz L, et al. New altered non-fibrillar collagens in human dilated cardiomyopathy: Role in the remodeling process. PLoS One. 2016;11(12):e0168130. doi: 10.1371/journal.pone.0168130 27936202 PMC5148085

[78] Tian Z, Miyata K, Kadomatsu T, Horiguchi H, Fukushima H, Tohyama S, et al. ANGPTL2 activity in cardiac pathologies accelerates heart failure by perturbing cardiac function and energy metabolism. Nat Commun. 2016;7:13016. doi: 10.1038/ncomms13016 27677409 PMC5052800

[79] Quiles JM, Narasimhan M, Mosbruger T, Shanmugam G, Crossman D, Rajasekaran NS. Identification of transcriptome signature for myocardial reductive stress. Redox Biol. 2017;13:568–80. doi: 10.1016/j.redox.2017.07.013 28768233 PMC5536881

[80] Yang K-C, Jay PY, McMullen JR, Nerbonne JM. Enhanced cardiac PI3Kα signalling mitigates arrhythmogenic electrical remodelling in pathological hypertrophy and heart failure. Cardiovasc Res. 2012;93(2):252–62. doi: 10.1093/cvr/cvr283 22038742 PMC3258651

[81] Al Kury LT, Chacar S, Alefishat E, Khraibi AA, Nader M. Structural and electrical remodeling of the sinoatrial node in diabetes: New dimensions and perspectives. Front Endocrinol (Lausanne). 2022;13:946313. doi: 10.3389/fendo.2022.946313 35872997 PMC9302195

[82] Fabritz L, Herzig S. Can T-type calcium channels make a change of heart after myocardial infarction? Fiction or fact, and for better or for worse?. Cardiovasc Res. 2011;91(3):373–5. doi: 10.1093/cvr/cvr177 21685207

[83] Sollis E, Mosaku A, Abid A, Buniello A, Cerezo M, Gil L, et al. The NHGRI-EBI GWAS Catalog: knowledgebase and deposition resource. Nucleic Acids Res. 2023;51(D1):D977–85. doi: 10.1093/nar/gkac1010 36350656 PMC9825413

[84] Li J, Song J, Jiang M-H, Zheng J-G, Gao S-P, Zhu J-H, et al. Interleukin-6 promoter polymorphisms and susceptibility to atrial fibrillation in elderly Han Chinese patients with essential hypertension. J Interferon Cytokine Res. 2012;32(11):542–7. doi: 10.1089/jir.2012.0033 22924939 PMC3493041

[85] Marcus GM, Whooley MA, Glidden DV, Pawlikowska L, Zaroff JG, Olgin JE. Interleukin-6 and atrial fibrillation in patients with coronary artery disease: data from the heart and soul study. Am Heart J. 2008;155(2):303–9. doi: 10.1016/j.ahj.2007.09.006 18215601 PMC2247366

[86] Wang Q, Richardson TG, Sanderson E, Tudball MJ, Ala-Korpela M, Davey Smith G, et al. A phenome-wide bidirectional Mendelian randomization analysis of atrial fibrillation. Int J Epidemiol. 2022;51(4):1153–66. doi: 10.1093/ije/dyac041 35292824 PMC9365635

[87] Wang X, Huang T, Jia J. Proteome-wide Mendelian randomization analysis identified potential drug targets for atrial fibrillation. J Am Heart Assoc. 2023;12(16):e029003. doi: 10.1161/JAHA.122.029003 37581400 PMC10492951

[88] Rosa M, Chignon A, Li Z, Boulanger M-C, Arsenault BJ, Bossé Y, et al. A Mendelian randomization study of IL6 signaling in cardiovascular diseases, immune-related disorders and longevity. NPJ Genom Med. 2019;4:23. doi: 10.1038/s41525-019-0097-4 31552141 PMC6754413

[89] Yang K-C, Yamada KA, Patel AY, Topkara VK, George I, Cheema FH, et al. Deep RNA sequencing reveals dynamic regulation of myocardial noncoding RNAs in failing human heart and remodeling with mechanical circulatory support. Circulation. 2014;129(9):1009–21. doi: 10.1161/CIRCULATIONAHA.113.003863 24429688 PMC3967509

[90] Li Z Ren W Guo. The role of PCD-related circRNA/miRNA/mRNA regulatory network in the HF with AF [Internet]. 2023 [cited 2024 Jan 1]. Available from: https://www.ncbi.nlm.nih.gov/geo/query/acc.cgi?acc=GSE235757

[91] Xu Y. Identification of a long non-coding RNA as a novel biomarker and potential therapeutic target for atrial fibrillation [Internet]. 2016. Available from: https://www.ncbi.nlm.nih.gov/geo/query/acc.cgi?acc=GSE64904 10.18632/oncotarget.7514 PMC4905440 26908457

[92] Brown SM, Larsen NK, Thankam FG, Agrawal DK. Fetal cardiomyocyte phenotype, ketone body metabolism, and mitochondrial dysfunction in the pathology of atrial fibrillation. Mol Cell Biochem. 2021;476(2):1165–78. doi: 10.1007/s11010-020-03980-8 33188453 PMC7878362

[93] Kovacic JC, Dimmeler S, Harvey RP, Finkel T, Aikawa E, Krenning G. Endothelial to mesenchymal transition in cardiovascular disease: JACC state-of-the-art review. J Am Coll Cardiol. 2019;73:190–209. doi: 10.1016/j.jacc.2018.09.089 30654892 PMC6865825

[94] Kato T, Sekiguchi A, Sagara K, Tanabe H, Takamura M, Kaneko S. Endothelial-mesenchymal transition in human atrial fibrillation. J Cardiol. 2017;69:706–11. doi: 10.1016/j.jjcc.2016.10.014 27938856

[95] Saljic A, Grandi E, Dobrev D. Tgf-β1-induced endothelial-mesenchymal transition: a potential contributor to fibrotic remodeling in atrial fibrillation?. J Clin Invest. 2022;132. doi: 10.1172/JCI161070 35775488 PMC9246376

[96] Varela M, Colman MA, Hancox JC, Aslanidi OV. Atrial heterogeneity generates re-entrant substrate during atrial fibrillation and anti-arrhythmic drug action: Mechanistic insights from canine atrial models. PLoS Comput Biol. 2016;12(12):e1005245. doi: 10.1371/journal.pcbi.1005245 27984585 PMC5161306

[97] Elliott J, Mainardi L, Rodriguez Matas J. Cellular heterogeneity and repolarisation across the atria: An in silico study. Med Biol Eng Comput. 2022;60:3153–68. doi: 10.1007/s11517-022-02640-x 36104609 PMC9537222

[98] Denham NC, Pearman CM, Caldwell JL, Madders GWP, Eisner DA, Trafford AW, et al. Calcium in the Pathophysiology of Atrial Fibrillation and Heart Failure. Front Physiol. 2018;9:1380. doi: 10.3389/fphys.2018.01380 30337881 PMC6180171

[99] Greiser M, Kerfant B-G, Williams GSB, Voigt N, Harks E, Dibb KM, et al. Tachycardia-induced silencing of subcellular Ca2+ signaling in atrial myocytes. J Clin Invest. 2014;124(11):4759–72. doi: 10.1172/JCI70102 25329692 PMC4347234

[100] Kolwicz SC Jr, Purohit S, Tian R. Cardiac metabolism and its interactions with contraction, growth, and survival of cardiomyocytes. Circ Res. 2013;113(5):603–16. doi: 10.1161/CIRCRESAHA.113.302095 23948585 PMC3845521

[101] Rennison JH, Li L, Lin CR, Lovano BS, Castel L, Wass SY, et al. Atrial fibrillation rhythm is associated with marked changes in metabolic and myofibrillar protein expression in left atrial appendage. Pflugers Arch. 2021;473(3):461–75. doi: 10.1007/s00424-021-02514-5 33454842 PMC7940600

[102] Tu T, Qin F, Bai F, Xiao Y, Ma Y, Li B. Quantitative acetylated proteomics on left atrial appendage tissues revealed atrial energy metabolism and contraction status in patients with valvular heart disease with atrial fibrillation. Front Cardiovasc Med. 2022;9:962036. doi: 10.3389/fcvm.2022.962036 36176981 PMC9513032

[103] Mason FE, Pronto JRD, Alhussini K, Maack C, Voigt N. Cellular and mitochondrial mechanisms of atrial fibrillation. Basic Res Cardiol. 2020;115(6):72. doi: 10.1007/s00395-020-00827-7 33258071 PMC7704501

[104] Lenski M, Schleider G, Kohlhaas M, Adrian L, Adam O, Tian Q, et al. Arrhythmia causes lipid accumulation and reduced glucose uptake. Basic Res Cardiol. 2015;110(4):40. doi: 10.1007/s00395-015-0497-2 26018791

[105] Friedrichs K, Adam M, Remane L, Mollenhauer M, Rudolph V, Rudolph TK, et al. Induction of atrial fibrillation by neutrophils critically depends on CD11b/CD18 integrins. PLoS One. 2014;9(2):e89307. doi: 10.1371/journal.pone.0089307 24558493 PMC3928425

[106] Rudolph V, Andrié RP, Rudolph TK, Friedrichs K, Klinke A, Hirsch-Hoffmann B, et al. Myeloperoxidase acts as a profibrotic mediator of atrial fibrillation. Nat Med. 2010;16(4):470–4. doi: 10.1038/nm.2124 20305660 PMC2880896

[107] Mołek P, Ząbczyk M, Malinowski KP, Natorska J, Undas A. Enhanced neutrophil extracellular traps formation in AF patients with dilated left atrium. Eur J Clin Invest. 2023;53(5):e13952. doi: 10.1111/eci.13952 36635213

[108] Lu M, Zhang Y, Liu R, He X, Hou B. Predictive value of neutrophil to lymphocyte ratio for ischemic stroke in patients with atrial fibrillation: A meta-analysis. Front Neurol. 2022;13:1029010. doi: 10.3389/fneur.2022.1029010 36578303 PMC9792176

[109] Liu Z, Nguyen Khuong J, Borg Caruana C, Jackson SM, Campbell R, Ramson DM, et al. The prognostic value of elevated perioperative neutrophil-lymphocyte ratio in predicting postoperative atrial fibrillation after cardiac surgery: A systematic review and meta-analysis. Heart Lung Circ. 2020;29:1015–24. doi: 10.1016/j.hlc.2019.11.021 32089488

[110] Shao Q, Chen K, Rha S-W, Lim H-E, Li G, Liu T. Usefulness of neutrophil/lymphocyte ratio as a predictor of atrial fibrillation: A meta-analysis. Arch Med Res. 2015;46(3):199–206. doi: 10.1016/j.arcmed.2015.03.011 25980945

