# Supplementary material for: The transcriptional landscape of atrial fibrillation: A systematic review and meta-analysis
Source: PLoS One. 2025 May 30;20(5):e0323534. doi: 10.1371/journal.pone.0323534 (PMC12124854; doi:10.1371/journal.pone.0323534)
Supplement: S1 Text — Search strategies used in each evaluated database. (DOCX) [file pone.0323534.s001.docx]

**Supplemental Text 1.** Search strategies used in each evaluated database.

**Medline**(Ovid),

(Atrial Fibrillation.mp OR Paroxysmal atrial fibrillation.mp OR "Atrial fibrillation and flutter".mp OR Chronic atrial fibrillation.mp OR Persistent atrial fibrillation.mp OR Permanent atrial fibrillation.mp OR Fibrillation atrial aggravated.mp) AND ((RNA-Seq/ OR Gene Expression Profiling/ OR Transcriptome/ OR High-Throughput Nucleotide Sequencing/ OR Oligonucleotide Array Sequence Analysis/) OR (Microarray Analysis/ OR Gene Expression Regulation/)) AND Humans/

**Embase**,

('atrial fibrillation':ab,ti,kw OR 'heart atrium arrhythmia':ab,ti,kw OR 'atrial cardiomyopathy':ab,ti,kw) AND ('gene expression':ab,ti,kw OR 'rna sequencing':ab,ti,kw OR 'microarray analysis':ab,ti,kw OR 'transcriptome sequencing':ab,ti,kw OR 'transcriptomic analysis':ab,ti,kw OR 'transcriptomic profiling':ab,ti,kw OR 'transcriptomics':ab,ti,kw) AND ([article]/lim OR [article in press]/lim OR [data papers]/lim) AND [2000-2023]/py

**CINAHL**(EBSCOhost),

("atrial fibrillation" or "atrial cardiomyopathy" or "atrial arrhythmia") AND ("rna-seq" or "rna sequencing" or transcriptomic* or microarray* or "high throughput sequencing" or "next generation sequencing" or "gene expression analysis")

**Google Scholar**

(atrial fibrillation or atrial cardiomyopathy or atrial arrhythmia) AND (rna-seq or rna sequencing or transcriptom* or microarray* or high throughput sequencing or next generation sequencing or gene expression analysis)
