# Supplementary material for: The transcriptional landscape of atrial fibrillation: A systematic review and meta-analysis
Source: PLoS One. 2025 May 30;20(5):e0323534. doi: 10.1371/journal.pone.0323534 (PMC12124854; doi:10.1371/journal.pone.0323534)
Supplement: S1 Fig — (DOCX) [file pone.0323534.s010.docx]

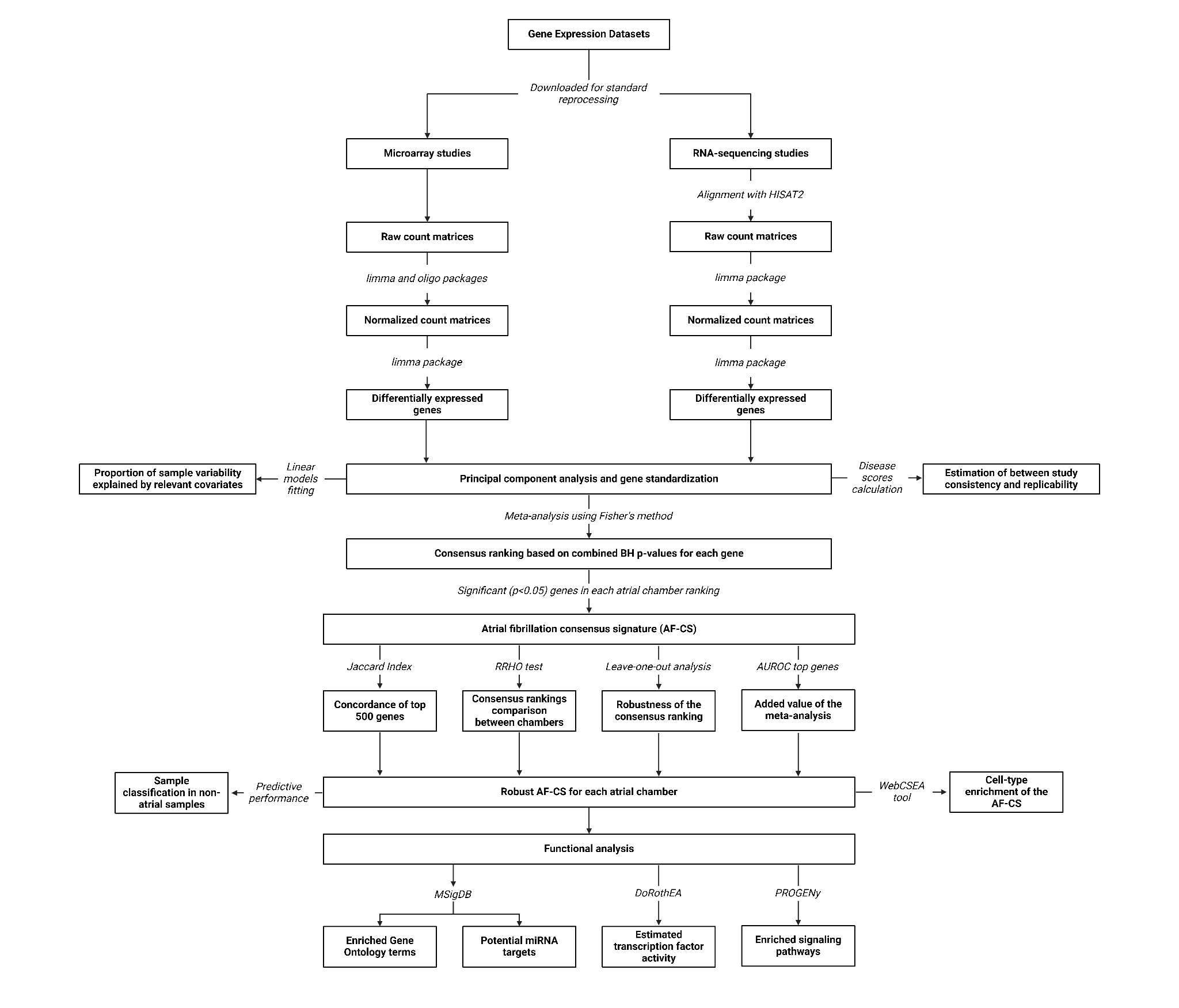


**Supplemental Figure 1.** Summary of the analysis process of the atrial fibrillation consensus signature meta-analysis.
