# Supplementary material for: The transcriptional landscape of atrial fibrillation: A systematic review and meta-analysis
Source: PLoS One. 2025 May 30;20(5):e0323534. doi: 10.1371/journal.pone.0323534 (PMC12124854; doi:10.1371/journal.pone.0323534)
Supplement: S2 Fig — A) Mean age and standard deviation of the age (years) in atrial fibrillation (AF) and sinus rhythm (SR) groups per study. B) Relative frequencies of each sex per study. C) Mean age and standard deviation of the LVEF (%) in AF and SR groups per study. (DOCX) [file pone.0323534.s011.docx]

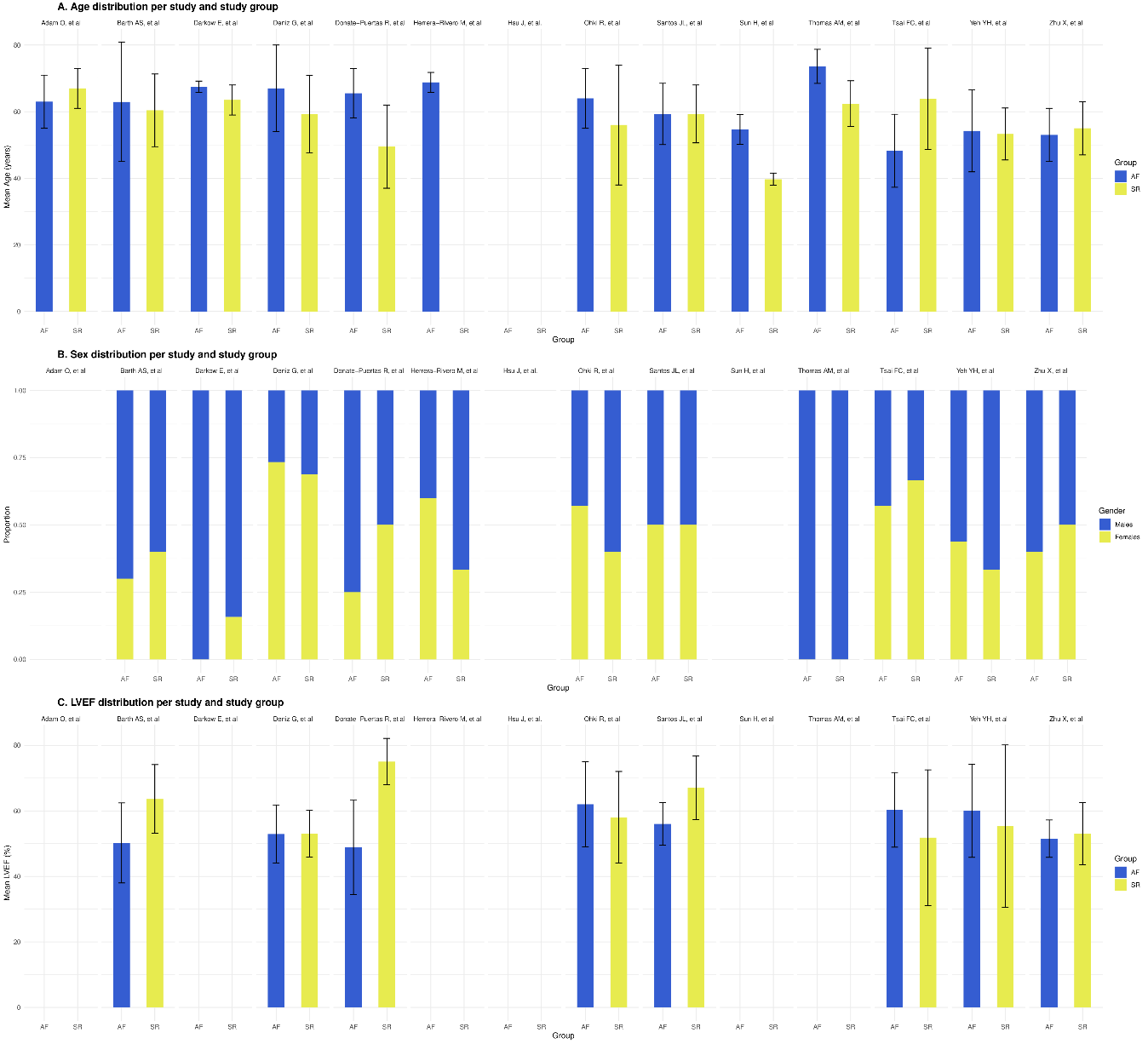


**Supplemental Figure 2.** Age, sex, and left ventricular ejection fraction (LVEF) distribution in the included studies. A) Mean age and standard deviation of the age (years) in atrial fibrillation (AF) and sinus rhythm (SR) groups per study. B) Relative frequencies of each sex per study. C) Mean age and standard deviation of the LVEF (%) in AF and SR groups per study.
