# Supplementary material for: The transcriptional landscape of atrial fibrillation: A systematic review and meta-analysis
Source: PLoS One. 2025 May 30;20(5):e0323534. doi: 10.1371/journal.pone.0323534 (PMC12124854; doi:10.1371/journal.pone.0323534)
Supplement: S3 Fig — A) Absolute gene coverage per study in the LAA-AF-CS. B) Pairwise comparison of covered genes in the LAA-AF-CS analysis using the Jaccard Index. C) Absolute gene coverage per study in the RAA-AF-CS. D) Pairwise comparison of covered genes in the RAA-CS analysis using the Jaccard Index. (DOCX) [file pone.0323534.s012.docx]

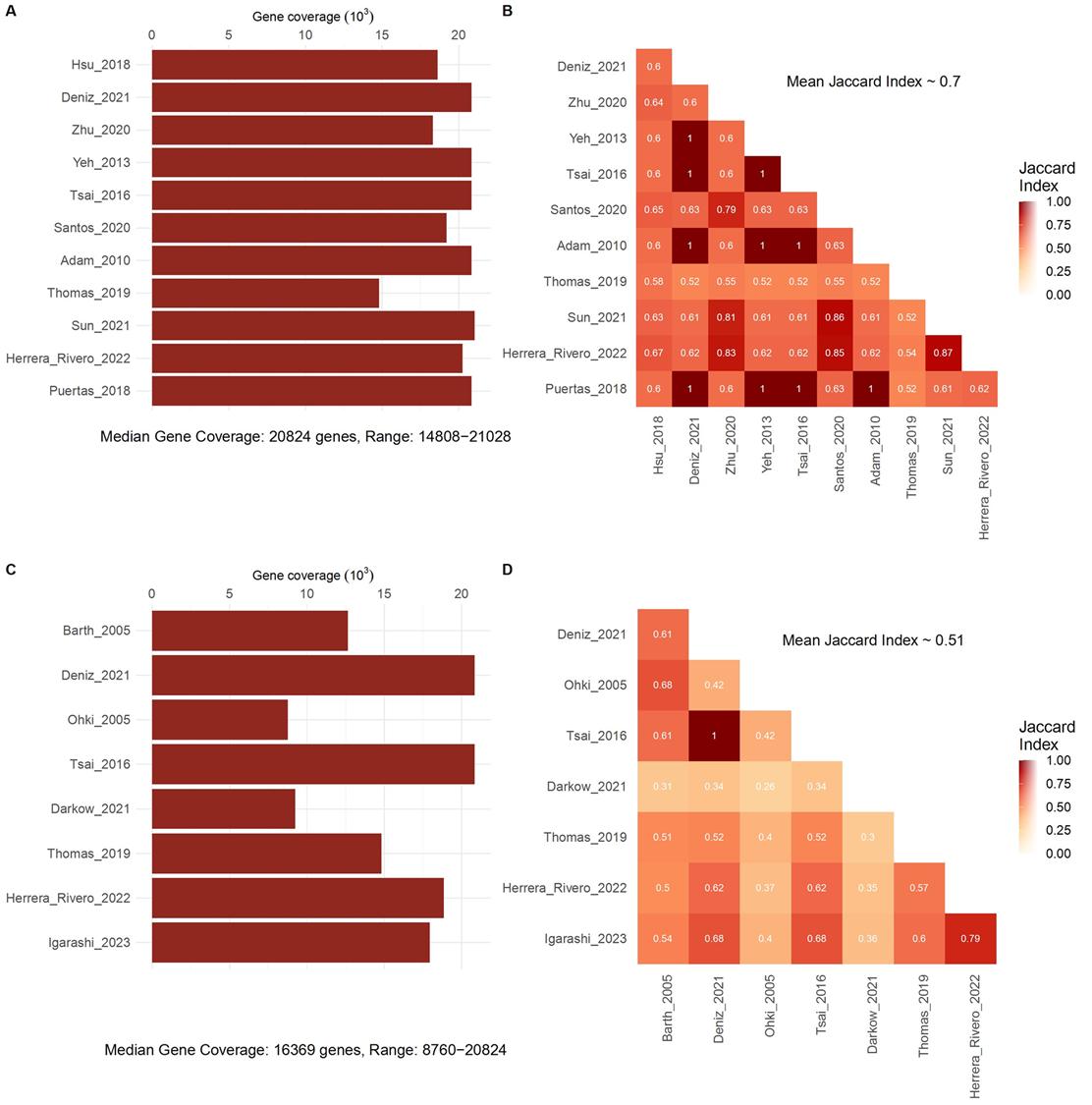


**Supplemental Figure 3.** Gene coverage summary and comparisons in included studies. A) Absolute gene coverage per study in the LAA-AF-CS. B) Pairwise comparison of covered genes in the LAA-AF-CS analysis using the Jaccard Index. C) Absolute gene coverage per study in the RAA-AF-CS. D) Pairwise comparison of covered genes in the RAA-CS analysis using the Jaccard Index.
