# Supplementary material for: The transcriptional landscape of atrial fibrillation: A systematic review and meta-analysis
Source: PLoS One. 2025 May 30;20(5):e0323534. doi: 10.1371/journal.pone.0323534 (PMC12124854; doi:10.1371/journal.pone.0323534)
Supplement: S4 Fig — A) PCA showing the first two components of the samples from the included studies in the LAA-AF-CS. B) PCA showing the first two components of the AF samples from the included studies in the LAA-AF-CS after z-transforming. C) t-distributed stochastic neighbor embedding (t-SNE) applied to standardized AF samples in the LAA-AF-CS. D) PCA showing the first two components of the samples from the included studies in the RAA-AF-CS. E) PCA showing the first two components of the AF samples from the included studies in the RAA-AF-CS after z-transforming. F) t-distributed stochastic neighbor embedding (t-SNE) applied to standardized AF samples in the RAA-AF-CS. (DOCX) [file pone.0323534.s013.docx]

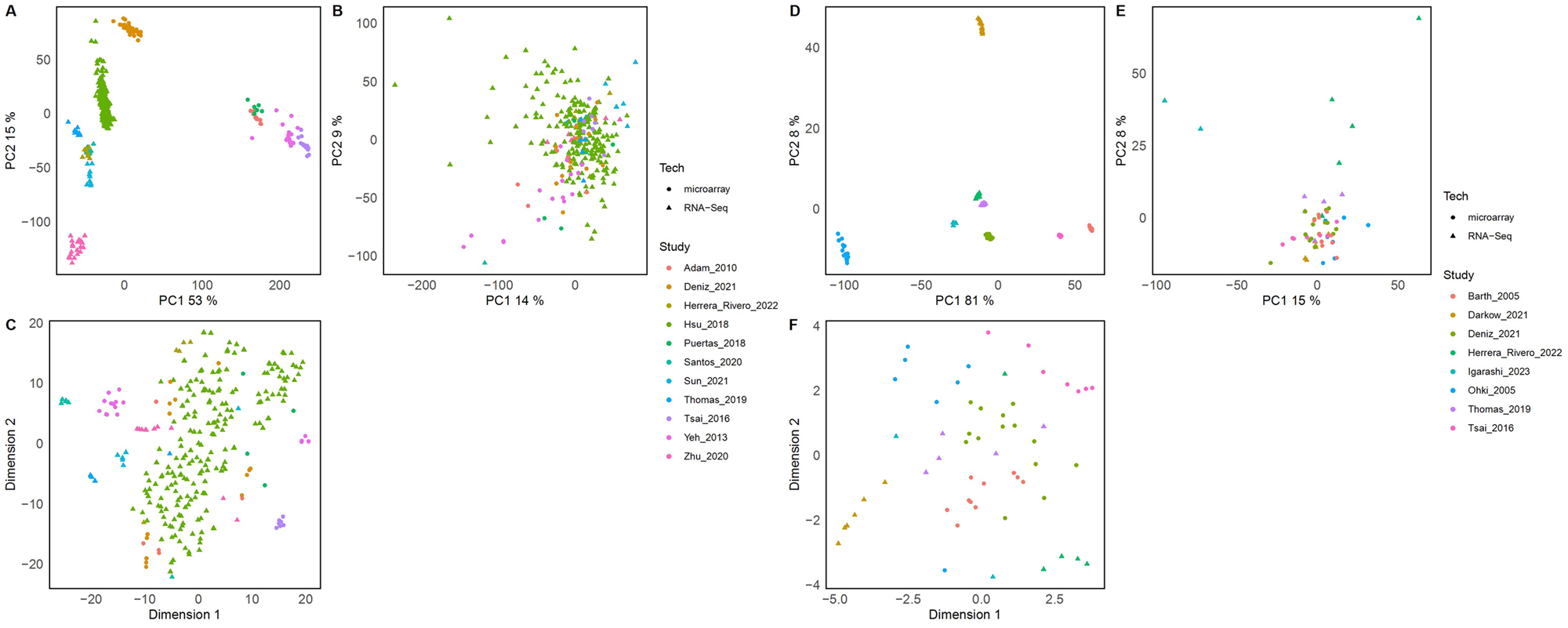


**Supplemental Figure 4.** Principal Component Analysis (PCA) of each individual sample in the included studies. A) PCA showing the first two components of the samples from the included studies in the LAA-AF-CS. B) PCA showing the first two components of the AF samples from the included studies in the LAA-AF-CS after z-transforming. C) t-distributed stochastic neighbor embedding (t-SNE) applied to standardized AF samples in the LAA-AF-CS. D) PCA showing the first two components of the samples from the included studies in the RAA-AF-CS. E) PCA showing the first two components of the AF samples from the included studies in the RAA-AF-CS after z-transforming. F) t-distributed stochastic neighbor embedding (t-SNE) applied to standardized AF samples in the RAA-AF-CS.
