# Supplementary material for: The transcriptional landscape of atrial fibrillation: A systematic review and meta-analysis
Source: PLoS One. 2025 May 30;20(5):e0323534. doi: 10.1371/journal.pone.0323534 (PMC12124854; doi:10.1371/journal.pone.0323534)
Supplement: S5 Fig — A) LAA-AF-CS. B) RAA-AF-CS. (DOCX) [file pone.0323534.s014.docx]

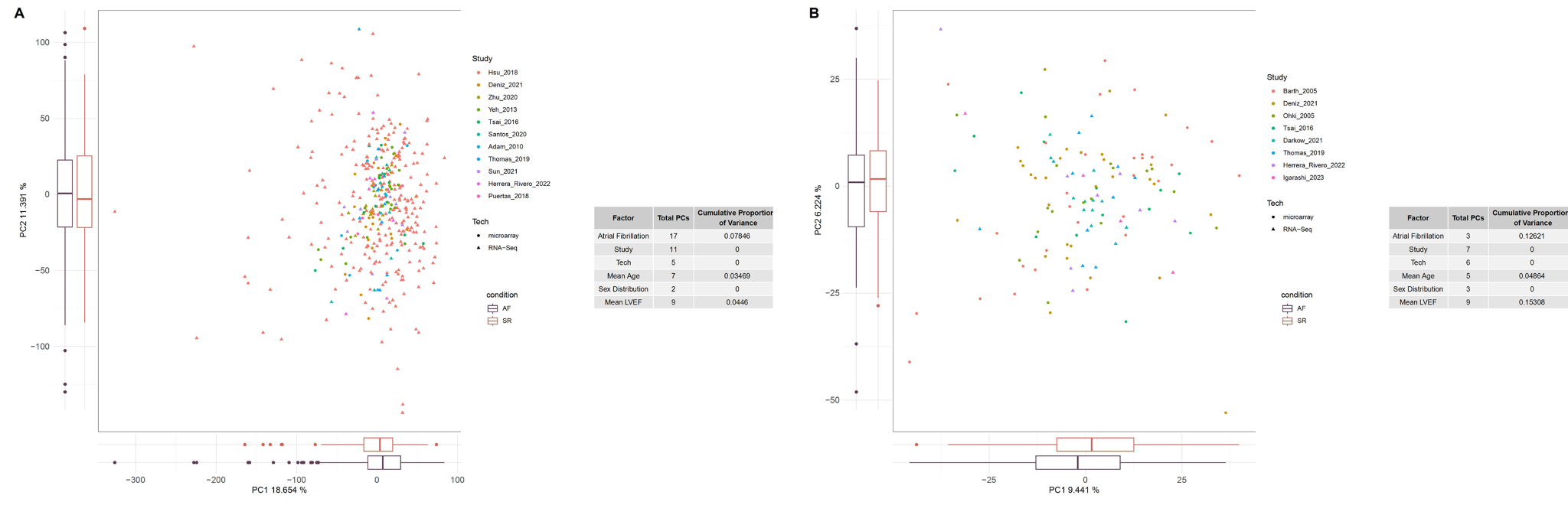


**Supplemental Figure 5.** PCA showing the first two components after gene standardization and the cumulative proportion of variance explained by components related to AF, study, technology used, age, sex, and LVEF. A) LAA-AF-CS. B) RAA-AF-CS.
