# Supplementary material for: The transcriptional landscape of atrial fibrillation: A systematic review and meta-analysis
Source: PLoS One. 2025 May 30;20(5):e0323534. doi: 10.1371/journal.pone.0323534 (PMC12124854; doi:10.1371/journal.pone.0323534)
Supplement: S6 Fig — A) LAA-AF-CS. B) RAA-AF-CS. (DOCX) [file pone.0323534.s015.docx]

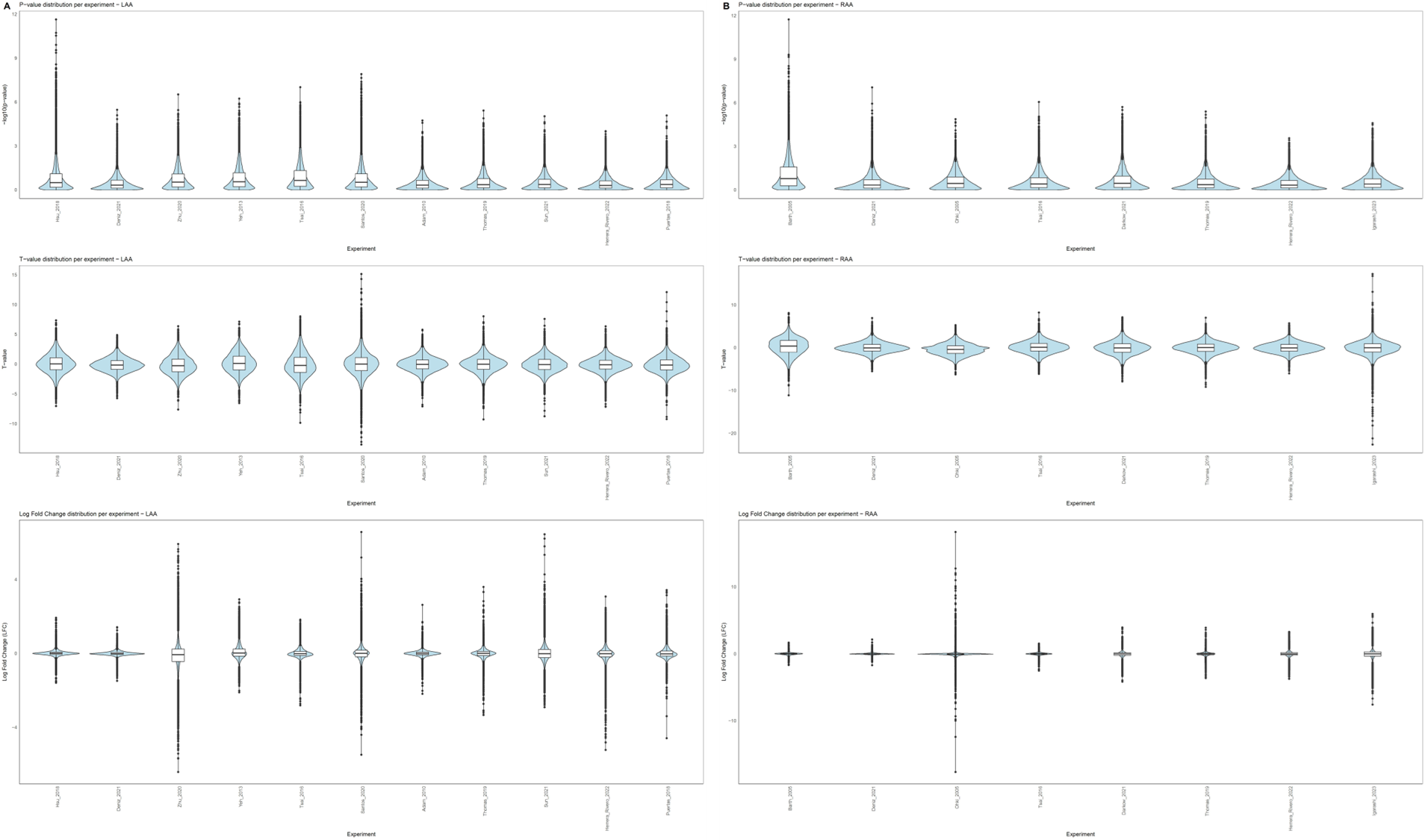


**Supplemental Figure 6.** Summary of distributions of -log10(p-values), t-values, and log2(fold-changes) derived from the differential expression analyses. A) LAA-AF-CS. B) RAA-AF-CS.
