# Supplementary material for: The transcriptional landscape of atrial fibrillation: A systematic review and meta-analysis
Source: PLoS One. 2025 May 30;20(5):e0323534. doi: 10.1371/journal.pone.0323534 (PMC12124854; doi:10.1371/journal.pone.0323534)
Supplement: S7 Fig — (DOCX) [file pone.0323534.s016.docx]

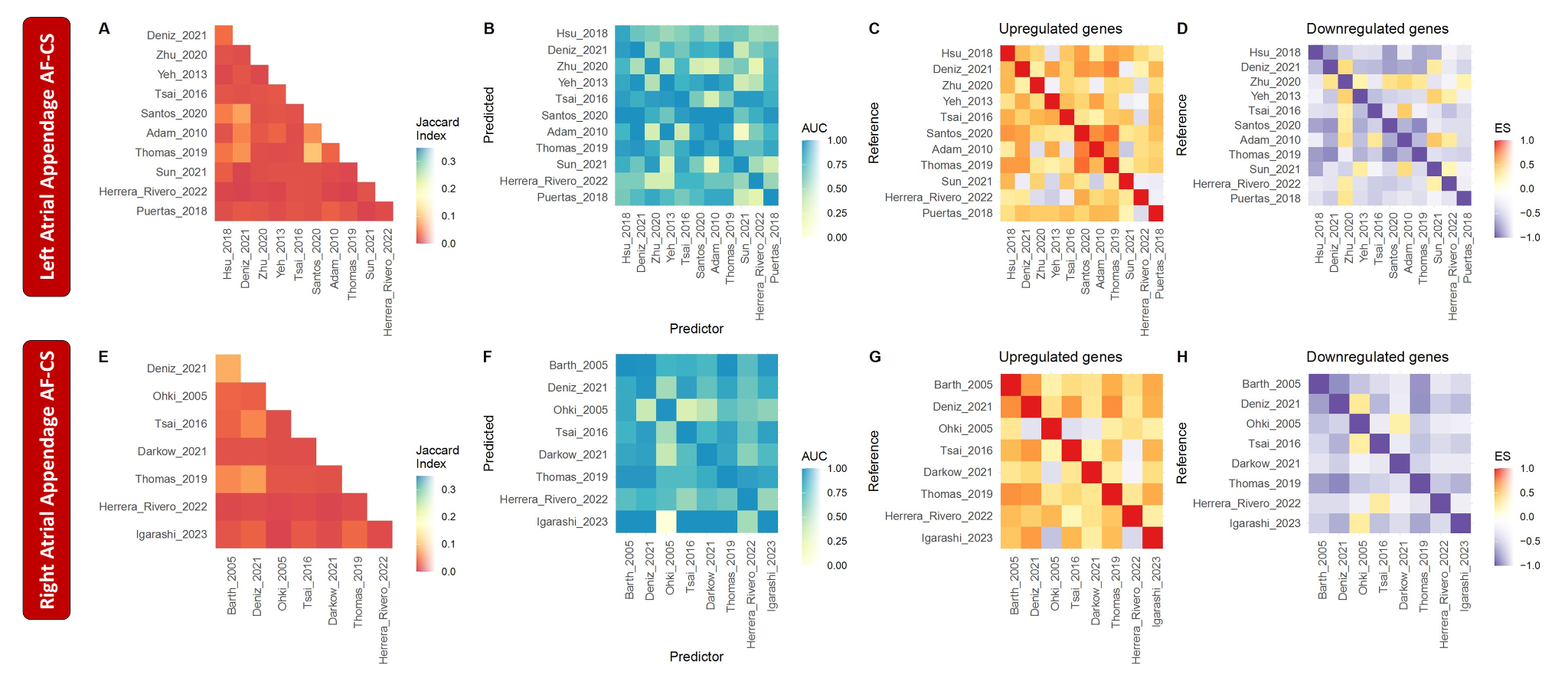


**Supplemental Figure 7.** Consistency of the transcriptomic signatures of AF across the included studies.

**A,** Contrasts of the top 500 differentially expressed genes (DEG) using the Jaccard index in the LAA-AF-CS. **B,** Contrasts of the top 500 differentially expressed genes (DEG) using the Jaccard index in the RAA-AF-CS. **C,** Area under the curve (AUC) of the performance of the studies to predict AF/SR status in other datasets by using a disease score composed of the top 500 DEG of each study in the LAA-AF-CS. **D,** Area under the curve (AUC) of the performance of the studies to predict AF/SR status in other datasets by using a disease score composed of the top 500 DEG of each study in the RAA-AF-CS. **E,** Enrichment score (ES) representing the degree to which each study's top 500 up-regulated DEG are over-represented at the top or bottom of a ranked gene list in each other dataset in the LAA-AF-CS. **F,** ES representing the degree to which each study's top 500 up-regulated DEG are over-represented at the top or bottom of a ranked gene list in each other dataset in the RAA-AF-CS. **G,** ES representing the degree to which each study's top 500 down-regulated DEG are over-represented at the top or bottom of a ranked gene list in each other dataset in the LAA-AF-CS. **H,** ES representing the degree to which each study's top 500 down-regulated DEG are over-represented at the top or bottom of a ranked gene list in each other dataset in the RAA-AF-CS.
