# Supplementary material for: The transcriptional landscape of atrial fibrillation: A systematic review and meta-analysis
Source: PLoS One. 2025 May 30;20(5):e0323534. doi: 10.1371/journal.pone.0323534 (PMC12124854; doi:10.1371/journal.pone.0323534)
Supplement: S8 Fig — A) Disease Score in the LAA-AF-CS. B) Enrichment Score in the LAA-AF-CS. C) Disease Score in the RAA-AF-CS. D) Enrichment Score in the RAA-AF-CS. (DOCX) [file pone.0323534.s017.docx]

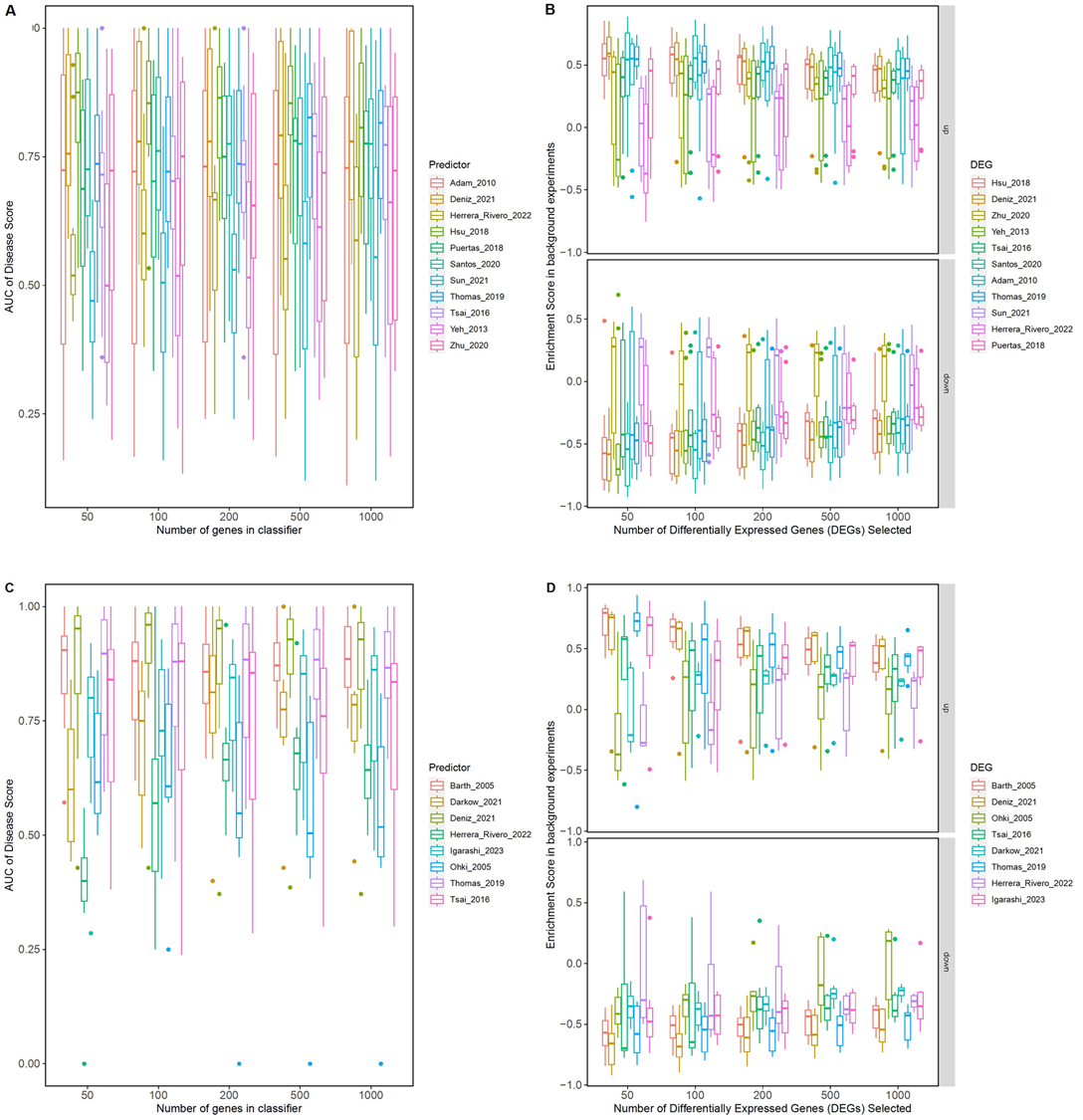


**Supplemental Figure 8.** Evaluation of the reliability of measures for comparing study results in the meta-analysis. A) Disease Score in the LAA-AF-CS. B) Enrichment Score in the LAA-AF-CS. C) Disease Score in the RAA-AF-CS. D) Enrichment Score in the RAA-AF-CS.
