# Supplementary material for: The transcriptional landscape of atrial fibrillation: A systematic review and meta-analysis
Source: PLoS One. 2025 May 30;20(5):e0323534. doi: 10.1371/journal.pone.0323534 (PMC12124854; doi:10.1371/journal.pone.0323534)
Supplement: S10 Fig — A) LAA-AF-CS. B) RAA-AF-CS. (DOCX) [file pone.0323534.s019.docx]

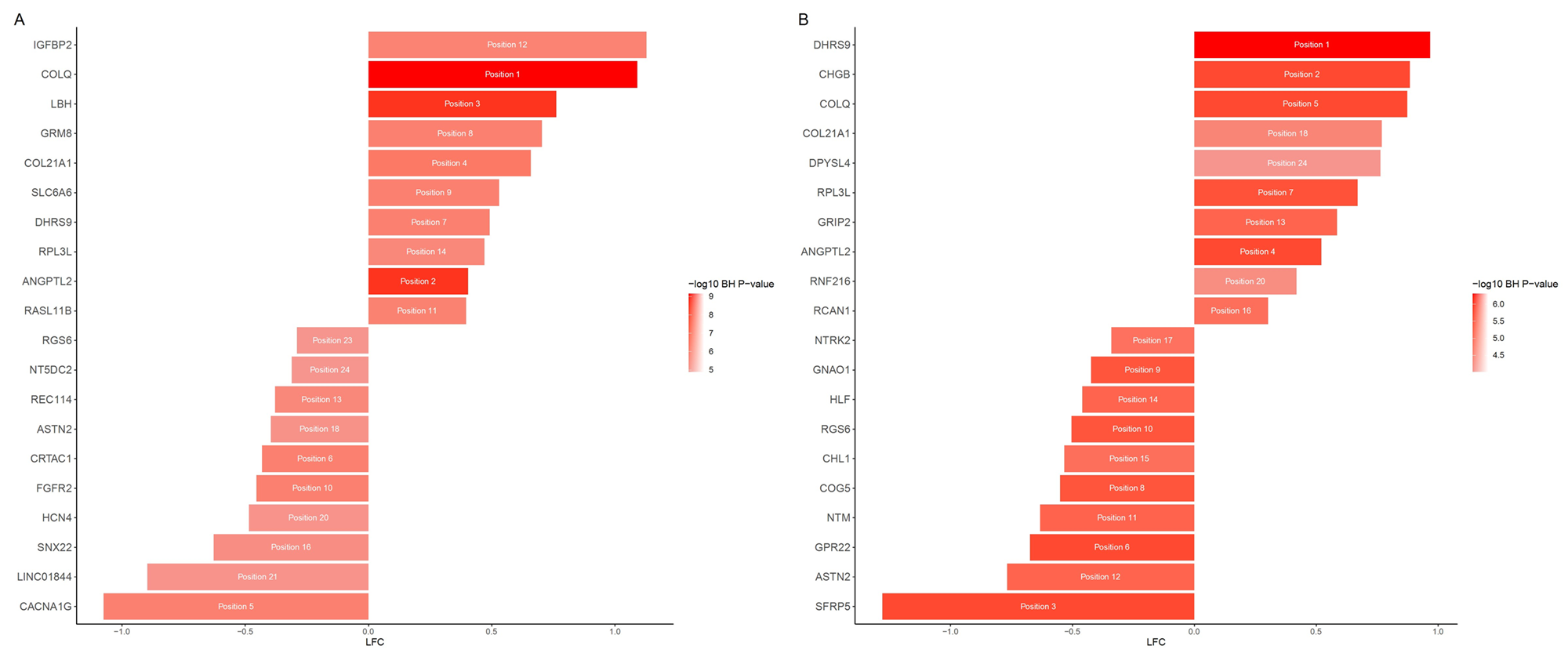


**Supplemental Figure 10.** Summary of the top-ranking genes according to log-fold change (LFC) and position in the consensus signature. A) LAA-AF-CS. B) RAA-AF-CS.
