# Supplementary figures and images for: The transcriptional landscape of atrial fibrillation: A systematic review and meta-analysis
Source: PLoS One. 2025 May 30;20(5):e0323534. doi: 10.1371/journal.pone.0323534 (PMC12124854; doi:10.1371/journal.pone.0323534)

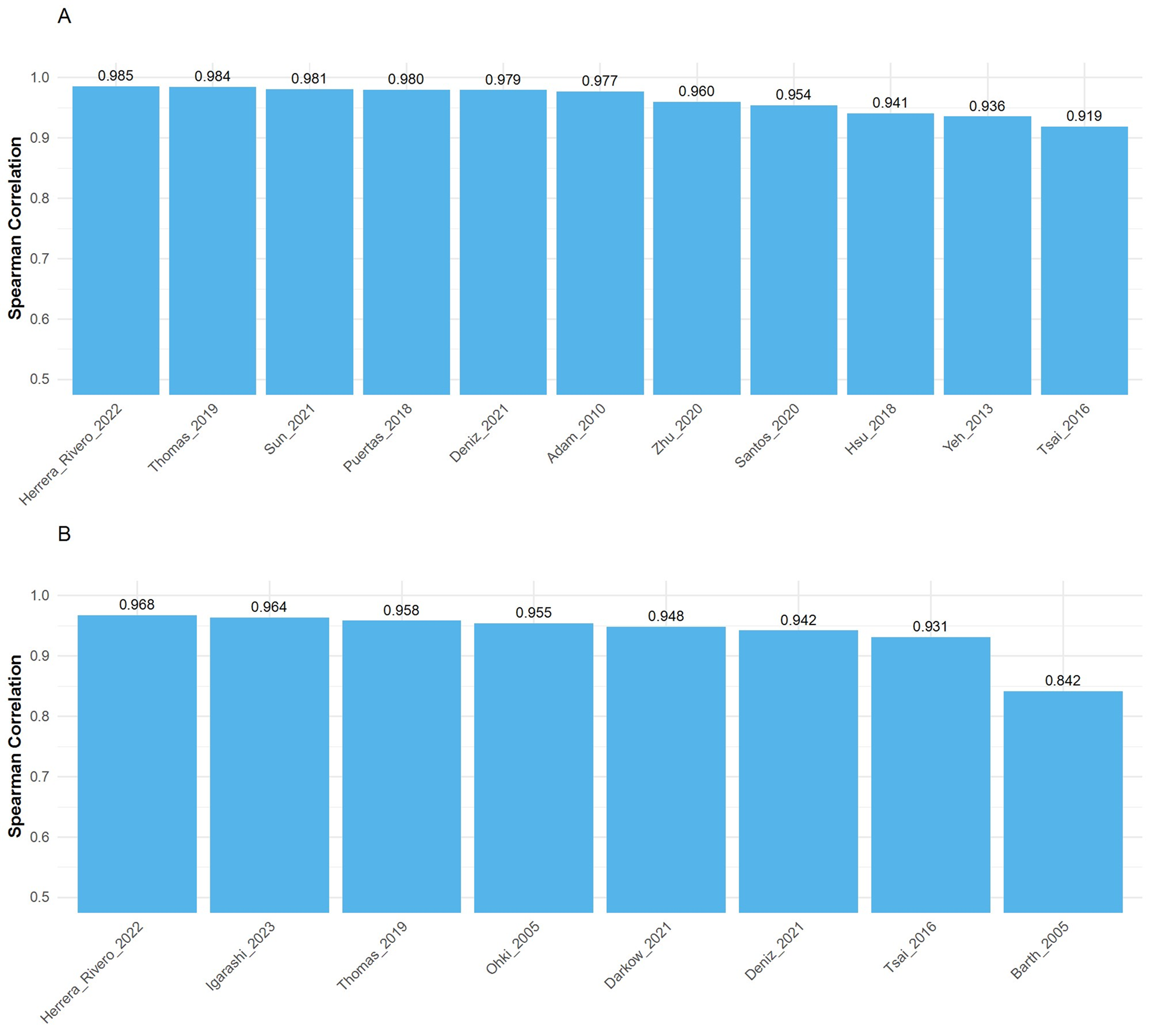


**Supplemental Figure 11.** Results of leave-one-out sensitivity analysis. A) LAA-AF-CS. B) RAA-AF-CS.

Supplement: S11 Fig — A) LAA-AF-CS. B) RAA-AF-CS. (DOCX) [file pone.0323534.s020.docx]
