# Supplementary material for: The transcriptional landscape of atrial fibrillation: A systematic review and meta-analysis
Source: PLoS One. 2025 May 30;20(5):e0323534. doi: 10.1371/journal.pone.0323534 (PMC12124854; doi:10.1371/journal.pone.0323534)
Supplement: S12 Fig — (DOCX) [file pone.0323534.s021.docx]

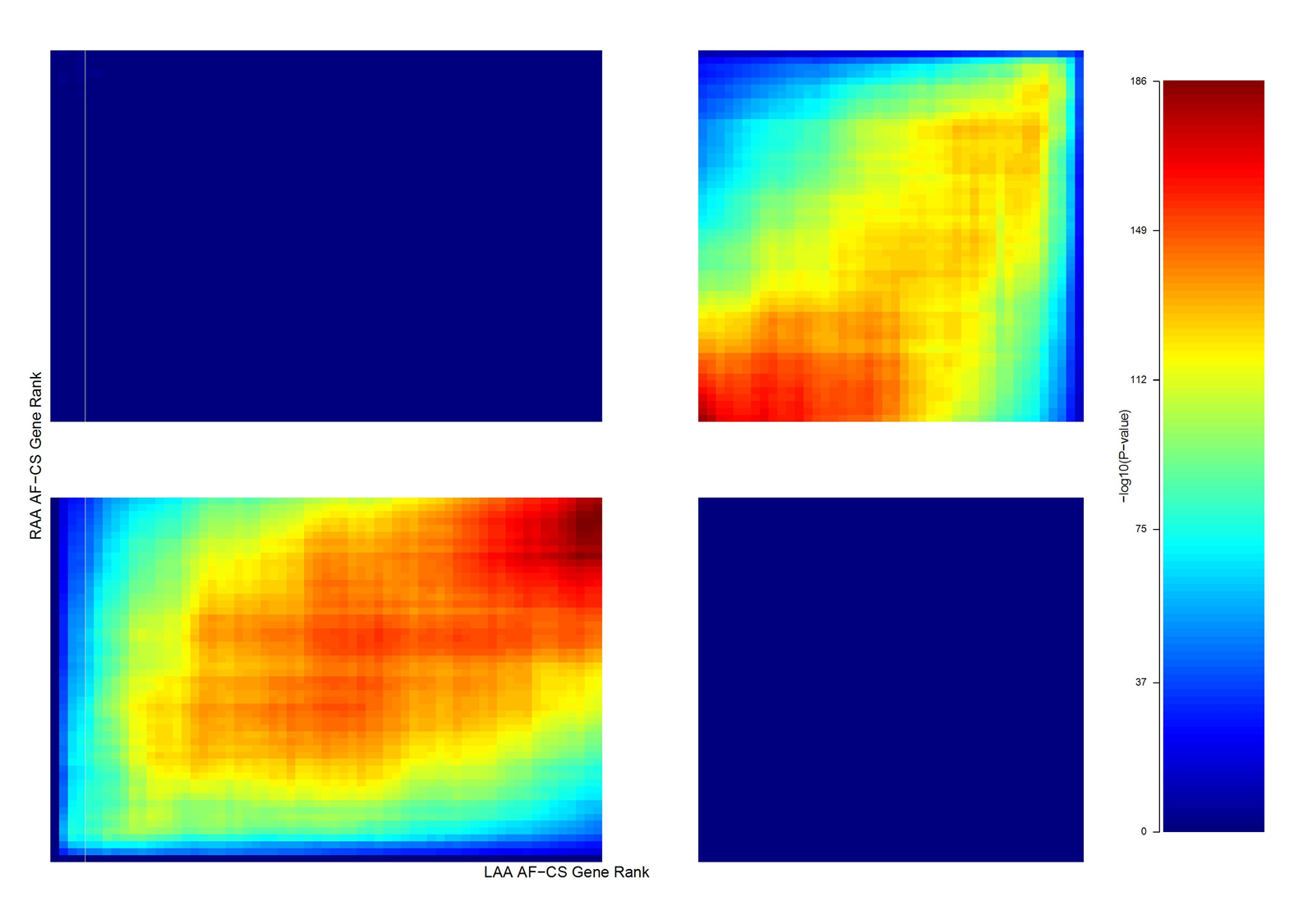


**Supplemental Figure 12.** Rank Rank Hypergeometric Overlap (RRHO) heatmap comparing the gene expression signatures from the LAA-AF-CS and RAA-AF-CS.
